# Favorable QTL Alleles for Yield and Its Components Identified by Association Mapping in Chinese Upland Cotton Cultivars

**DOI:** 10.1371/journal.pone.0082193

**Published:** 2013-12-26

**Authors:** Hongxian Mei, Xiefei Zhu, Tianzhen Zhang

**Affiliations:** 1 National Key Laboratory of Crop Genetics & Germplasm Enhancement, Cotton Research Institute, Nanjing Agricultural University, Nanjing, China; 2 Sesame Research Center, Henan Academy of Agricultural Sciences, Zhengzhou, China; Nanjing Forestry University, China

## Abstract

Linkage disequilibrium based association mapping is a powerful tool for dissecting the genetic basis underlying complex traits. In this study, an association mapping panel consisting of 356 representative Upland cotton cultivars was constructed, evaluated in three environments and genotyped using 381 SSRs to detect molecular markers associated with lint yield and its components. The results showed that abundant phenotypic and moderate genetic diversities existed within this germplasm panel. The population could be divided into two subpopulations, and weak relatedness was detected between pair-wise accessions. LD decayed to the background (*r* ^2^ = 0.1182, P≤0.01), *r* ^2^ = 0.1 and *r* ^2^ = 0.2 level within 12–13 cM, 17–18 cM and 3–4 cM, respectively, providing the potential for association mapping of agronomically important traits in Chinese Upland cotton. A total of 55 marker-trait associations were detected between 26 SSRs and seven lint yield traits, based on a mixed linear model (MLM) and Bonferroni correction (*P*≤0.05/145, −log_10_ *P*≥3.46). Of which 41 could be detected in more than one environment and 17 markers were simultaneously associated with two or more traits. Many associations were consistent with QTLs identified by linkage mapping in previous reports. Phenotypic values of alleles of each loci in 41 stably detected associations were compared, and 23 favorable alleles were identified. Population frequency of each favorable allele in historically released cultivar groups was also evaluated. The QTLs detected in this study will be helpful in further understanding the genetic basis of lint yield and its components, and the favorable alleles may facilitate future high-yield breeding by genomic selection in Upland cotton.

## Introduction

Cotton is the most important natural textile fiber source globally. The worldwide economic impact of the cotton industry is estimated at approximately $500 billion per year with an annual utilization of about 27 million metric tons of cotton fiber. In recent years, demand for cotton fiber in the world market has dramatically increased, stock and use ratio dropped to 37% in 2010, compared to 55% in 2009. While cotton acreage has declined worldwide in the past few years, mainly due to strong competition from other crops as well as production costs (National Cotton Council, USA, http://www.cotton.org, 2012). The tetraploid species *Gossypium hirsutum* L. (n = 26, AD genome), commonly referred to as Upland cotton, accounts for 95% of the world's cotton production [1]. Thus, improving lint yield of Upland cotton cultivars will be critical for meeting worldwide demand, and maintaining profitability for cotton growers.

Lint yield is a complex trait in cotton, which is controlled by a large number of quantitative loci (QTLs). It is becoming progressively more difficult to improve lint yield using conventional breeding methods. Fortunately, the development in applied genomics research has provided alternative tools to improve efficiency in plant breeding programs. Molecular markers linked to the causal genes and QTLs can be used for marker-assisted selection (MAS) and/or genomic selection (GS) [2]–[3]. In the past two decades, a large number of QTLs for lint yield and fiber quality traits have been identified in Upland cotton [4]–[13]. However, approximately 80% of the previously reported QTLs could not be confirmed in subsequent studies, and few have actually been applied in breeding programs [14]–[16]. This may be because that most QTLs were population-specific, and the genetic variation detected in a unique bi-parental population might not be shared with other genetic populations, or shared but fixed in the parental lines. In addition, the limited genetic recombinations in most populations used for linkage mapping make it difficult to map QTLs with a high resolution, which severely limits their application in breeding programs. With the potential to exploit all recombination events that occurred in the evolutionary history of natural populations, linkage disequilibrium (LD) based association mapping (AM) has become a powerful approach for the dissection of complex traits and identification of causal variation with modest effects for target traits in many plant species [17]–[18] including cotton [19]–[24]. While the key constraints for the successful use of association mapping in plants are population structure and genetic relatedness, which can result in spurious marker-trait associations that may make it difficult to distinguish loci that truly affect the target traits [25]–[26]. Several statistical strategies have been developed to account for issues related to population structure and relatedness [27]–[29]. One powerful strategy is the unified mixed model approach (mixed linear model, MLM), which accounts for multiple levels of relatedness simultaneously, and can improve control of both type I and type II error rates [28]. In cotton, the first attempt of association mapping was reported by Kantartzi and Stewart in 2008. In that study, 30 marker and fiber trait associations were detected in 56 *Gossypium arboreum* accessions genotyped by 98 SSR markers [19]. Abdurakhmonov et al. performed an association mapping study, with the MLM model considering both kinship (K) and population structure (Q), of fiber quality traits by using a set of 95 core microsatellite markers in 285 exotic *Gossypium hirsutum* accessions and detected between 6% and 13% of SSR markers associated with the main fiber traits. Meanwhile, they indicated the genome-wide LD (r^2^≥0.1) declined at <10 cM in the landrace stocks and >30 cM in variety germplasm, but at r^2^≥0.2 which reduced to ∼1–2 cM and ∼6–8 cM, respectively [20]. Abdurakhmonov et al. performed another association mapping study of fiber quality traits using 202 microsatellite markers in a panel of 335 *G. hirsutum* varieties [21]. The result showed that the genome-wide LD extended up to 25 cM at r^2^≥0.1 and reduced to ∼5–6 cM at r^2^≥0.2 and an average of ∼20 SSR markers was associated with each main fiber quality trait in two environments. Zeng et al. carried out an association mapping study between 86 SSR markers and fiber traits using an exotic germplasm population of 260 lines derived from multiple crosses among *Gossypium* tetraploid species and found 59 markers were significantly associated with six fiber traits [22]. All the results mentioned above provided useful evidences of the potential for association mapping of agronomically important traits in cotton. But till now, association mapping study of lint yield traits has not been reported in cotton.

Although AM has been successfully used to detect the QTLs underlying quantitative traits in some crops, from a breeding standpoint, detecting associated loci is just the first step; analyzing the genetic effects of alleles and identifying favorable alleles will be more beneficial for target trait improvement. Breseghello & Sorrells identified several potentially beneficial alleles for kernel size and milling quality by comparing the average phenotypic value with specific alleles and null alleles in a soft winter wheat population [30]. Jia et al. identified some putative resistant alleles for Sheath Blight resistance in a rice panel composed of 217 accessions from the USDA core collection, and found that the number of putative resistant alleles presented in an entry was highly and significantly correlated with the decrease of ShB rating [31]. We performed a preliminary AM study in 81 Upland cotton cultivars and identified some elite alleles for yield and fiber quality traits [24]. China is the world's largest cotton-growing nation, but not a cotton domestication region. Most Upland cotton cultivars developed in China were derived from a few germplasm resources such as Deltapine (DPL), Stoneville (STV), Foster, King and Uganda, all of which were introduced from abroad [32]. Current and obsolete cultivars have been and continue to be the main resources for cotton breeding programs. Dissecting the genetic basis of lint yield and quality traits will be of great benefits to germplasm evaluation and future molecular breeding. In the present study, we aimed to detect QTLs underlying lint yield and its components, and to identify the favorable alleles in an AM panel composed of 356 accessions. We also analyzed genetic diversity, LD decay, population structure, genetic relatedness and favorable allele frequency in historically released cutivar groups. Our results should provide useful information for further understanding the genetic basis of lint yield and its components, and will facilitate future high-yield breeding by genomic selection in Upland cotton.

## Materials and Methods

### Association mapping panel construction

A total of 356 representative Upland cotton cultivars and breeding lines were selected from the cotton germplasm collections in our laboratory and the Cotton Research Institute, Chinese Academy of Agricultural Sciences (CRI-CAAS), and assembled to construct an AM panel. The population consisted of 348 cultivars developed in China, seven introduced from the U.S., including the genetic standard line TM-1, and one introduced from Uganda. According to their release year, the 348 Chinese cultivars could be divided into the following six groups: I (1930–1960, 26 lines); II (1961–1970, 26 lines); III (1971–1980, 39 lines), IV (1981–1990, 83 lines); V (1991–2000, 125 lines); and VI (2000–2005, 49 lines). The cultivars introduced from abroad, DPL 15, DPL 16, STV 2B, King, Foster 6 and Uganda 3 were used as a check group for genetic diversity and allele transmission evaluation, because they had been used as the main founder parents in China's Upland cotton breeding programs and are the progenitors of many cultivars [32]. All accessions have been self-pollinated for more than six generations and their detailed information are summarized in Table S1.

### Trait phenotyping

All of the accessions were planted in the following three environments to evaluate phenotypic performance: (1) Jiangpu Breeding Station, Nanjing Agricultural University, Nanjing, in 2009 (designated as E1), (2) Dafeng Agronomy Farm, Yancheng, Jiangsu Province (E2), and (3) Zhengzhou Agricultural Research Institute, Zhengzhou, Henan in 2010 (E3). The first two locations were in the Yangtze River cotton-growing region, and the third was in the Yellow River region. A randomized complete block design with single row plot and two replications was used in all field trails. The sowing dates were from late March to early April in different years and locations, and seedlings having up to 3–4 leaves were transplanted from seedbeds to fields, with 20 plants per row, a 30 cm plant-to-plant spacing, and 80 cm between rows. For most of the accessions are non-BT cottons, chemical control were used for preventing from bollworm damage and field managements were adjusted to local practices.

Field planting has been approved by Nanjing Agric Univ.. No specific permissions were required for these locations/activities since they are pure-line cultivars and the field studies did not involve endangered or protected species.

Ten consecutive plants in the middle of each row were tagged for trait measurement. Yield traits evaluated included: lint yield (LY, g/plant), seed cotton yield (SY, g/plant), bolls per plant (BN), boll weight (BW, g), lint percentage (LP, %), lint index (LI, g/100 seeds) and seed index (SI, g/100 seeds).

### SSR genotyping

Young leaves from each of the 356 accessions were collected and stored at −20°C. Total genomic DNA was extracted from the leaf samples as described by Guo et al. [33]. Based on the dense genetic linkage map constructed in our laboratory [33], 381 pairs of SSR primers that amplify loci evenly covering the tetraploid cotton genome (one marker per 10 cM, 186 on At and 195 on Dt subgenome with an average of 14.65 markers each chromosome) were selected to genotype the 356 accessions. The procedure for PCR-amplification and product analysis followed the published methods from our laboratory [34]–[35]. Since *G. hirsutum* is an allopolyploid species, SSR markers often yield complex band patterns and some of them had been located to more than one locus. To measure the complex band patterns of large scale genotypes, the band pattern in TM-1 (genetic standard line, one parent with which the reference linkage map was constructed) was treated as a check and the following criteria were used to assign the alleles to the corresponding loci: 1) when only one fragment was amplified in each accession, the fragments were regarded as alleles belonging to the single locus; 2) when multiple fragments were amplified in each line and the bands showed an obvious co-segregating relationship among different samples, they were regarded as alleles belonging to the same locus; and 3) when multiple bands produced in each line did not co-segregate among different accessions, the corresponding fragments in TM-1 that had been mapped to the reference map and co-segregated among different accessions were measured, other bands were discarded. According to the above criteria, the band pattern in TM-1 was designated as 1, the same patterns were also designated as 1, and the different ones were designated as 2, 3, 4, 5 and so on, thus the alleles from all accessions on each locus were measured. Markers with more than 10% missing data were not used in further analysis.

### Genotypic data analysis

Summary statistics including the total number of alleles, the number of alleles per locus, and gene diversity values were calculated using the software PowerMarker 3.25 [36]. The Bayesian model-based program STRUCTURE 2.3 was used to infer the population structure using 66 unlinked or weakly linked SSR markers [37]. The length of the burn-in period and the number of Markov Chain Monte Carlo replications after burn-in were all assigned at 100,000 with an admixture and allele frequencies correlated model. Five independent run iterations were performed with the hypothetical number of subpopulations (*k*) ranging from 1 to 10. The correct estimation of *k* was provided by joining the log probability of data [LnP(D)] from the STRUCTURE output and an ad hoc statistic *Δk* [38]. Based on the correct *k*, each accession was assigned to a subpopulation for which the membership value (Q value) was >0.5 [39], and the population structure matrix (Q) was generated for further marker-trait association mapping. The software SPAGeDi was used to calculate the pair-wise relatedness coefficients (K, kinship matrix) in order to estimate the genetic relatedness among individuals, with the negative value of kinship set to zero [40]. To estimate LD pattern in Upland cotton genome, the weighted average of squared correlation coefficient *r* ^2^ of each pair of SSR loci was calculated using the software package TASSEL 2.1 with rare alleles (allele frequency less than 0.05) treated as missing data [41]. The *r* ^2^ was estimated for total, linked and unlinked markers both in the entire panel and each subpopulation, respectively. The 99th percentile of *r* ^2^ distribution for unlinked markers, which determined whether LD is due to physical linkage [42], was treated as the background LD level [43].The *r^2^* values of each pair of SSR loci were plotted against map distance (cM), and LD decay was estimated.

### Phenotypic data analysis

Statistical analysis of all phenotypic data across three environments was performed with SAS 8.0 software (SAS Institute 1999). Analysis of variance (ANOVA) of all phenotypic data was calculated with PROC GLM, based on the trait means for each line across the three environments. Decomposition of variance components (genotype, environment, block, and the interactions among these factors) was evaluated using PROC VARCOMP, and the broad-sense heritability (*h*_B_ *^2^*) of each trait was estimated with the variance components. Correlation coefficients between traits were calculated with PROC CORR.

### Association mapping and favorable allele identification

Because the MLM model accounts for the effects both of population structure and genetic relatedness, and can significantly reduce spurious associations [28], the marker-trait AM was carried out with the MLM model as implemented in TASSEL software, and the *P* value and *R^2^* for each marker-trait association were determined [41]. Based on the results of AM, QTL alleles of loci significantly associated with the target traits were further analyzed. The phenotypic allele effect was estimated through comparison between the average phenotypic value over accessions with the specific allele and that of all accessions:
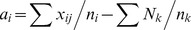
where *a_i_* is the phenotypic effect of the *ith* allele; *x_ij_* is the phenotypic value over the *jth* accession with the *ith* allele; *n_i_* is the number of accessions with the *ith* allele; *N_k_* is the phenotypic value over all accessions; *n_k_* is the number of accessions. If the value of *a_i_*>0, the allele is considered to have a positive effect, if it is <0, it corresponds to a negative allele. The favorable alleles were then identified according to the breeding objective of each target trait [24].

## Results

### Genetic diversity, population structure and genetic relatedness

Of the 381 SSR markers selected, only 145 amplified polymorphism (67 of 186 in At and 78 of 195 in Dt subgenome) in the present panel and a total of 415 alleles were detected (Table S2). The allele number, gene diversity and polymorphism information content (PIC) value of the 145 loci averaged 2.86, 0.32 and 0.27, respectively; with ranges of 2–9, 0.01–0.73 and 0.27–0.68, respectively. Approximately 80% of the polymorphic loci (115 of 145) had only two or three alleles. Among the 415 alleles detected, population frequencies of 131 alleles were rare (less than 0.05) and 34 were unique (detected in only one accession). The total number of alleles and the number of alleles per locus detected in the six historically released cultivar groups were much greater than that in the six founder parents ().

**Figure 1:**
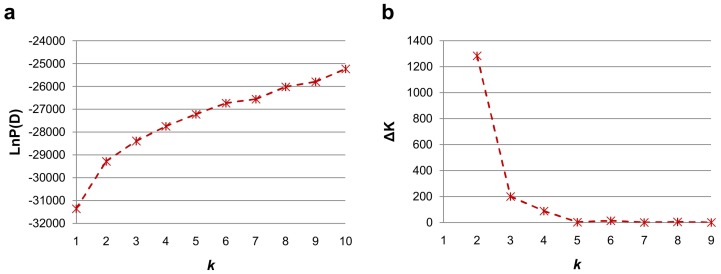
Estimated LnP(D) and Δ*K* over five repeats of STRUCTURE analysis. (a) LnP(D) for *k* from 1 to 10 for 356 accessions. LnP(D) value of each hypothetical *k* kept increasing with *k* value and did not show any peak. (b) Δ*K* for *k* from 2 to 9 for 356 accessions. The *Δk* value showed a much higher likelihood at *k* = 2 than at *k* = 3–10, suggesting that the total panel should be divided into two major subpopulations.

The model-based evaluation of the population structure of the 356 Upland cotton cultivars showed that the LnP(D) value corresponding to each hypothetical *k* kept increasing with *k* value and did not show any peak. The *Δk* value showed a much higher likelihood at *k* = 2 than at *k* = 3–10 (**Figure 1**), suggesting that the total panel could be divided into two major subpopulations [38], designated as P1 and P2, respectively. The P1 group contained 115 accessions including 63 cultivars from Yellow River cotton growing region, 46 lines from North and Northwest China regions, and six cultivars from Yangtze River region. The P2 group consisted of 241 accessions including 116 lines from Yellow River cotton growing region, 107 lines from Yangtze River region, 10 lines from the North and Northwest China regions, and eight lines intrduced from abroad (Table S1). Then, the corresponding Q matrix at *k* = 2 was used for the following association analysis.

For the kinship coefficient values, 86.85% was less than 0.05, 8.56% had a range of 0.05–0.10, and the remaining 4.59% showed various degrees of genetic relatedness (data not shown). Based on the results of the relatedness analysis, a K matrix was constructed for association mapping.

### Pair-wise linkage disequilibrium across the whole genome

The *r* ^2^ was calculated for total, linked and unlinked markers (**Table 1**), respectively, with SSR loci on the same chromosome considered as linked and those from different chromosomes as unlinked. In the entire panel, the average *r* ^2^ of locus pairs was 0.0103, and 18.29% were significant (P≤0.01). Moreover, 21.03% of the linked locus pairs and 18.18% of the unlinked pairs showed significant LD (P≤0.01) with the average *r* ^2^ of 0.0160 and 0.0101, respectively. In the subpopulation P1 and P2, the average *r* ^2^ of locus pairs was 0.0151 and 0.0104, respectively, and the proportion of significant LD (P≤0.01) was 5.10% and 10.78%, respectively. In the entire panel and subpopulations, both average *r* ^2^ and proportion of significant LD for linked loci were all higher than those for unlinked markers (**Table 1**).

**Table 1:** LD in the entire panel and subpopulations.

Groups^a^	Total^b^		Linked^c^		Unlinked^d^	
	*r* ^2^	Sig. LD (%)^e^	*r* ^2^	Sig. LD (%)^e^	*r* ^2^	Sig. LD (%)^e^
P1	0.0151	5.10	0.0194	7.31	0.015	5.02
P2	0.0104	10.78	0.0172	11.11	0.0101	10.77
Entire panel	0.0103	18.29	0.0160	21.03	0.0101	18.18

^a^ Groups P1 and P2 were classified based on the results of STRUCTURE analysis of the 356 Upland cotton accessions.

^b^ The total set of locus pairs, including linked and unlinked loci.

^c^ Pairs of loci on the same chromosome.

^d^ Pairs of loci from different chromosomes.

^e^ Significant threshold is set to P≤0.01.

**Figure 2:**
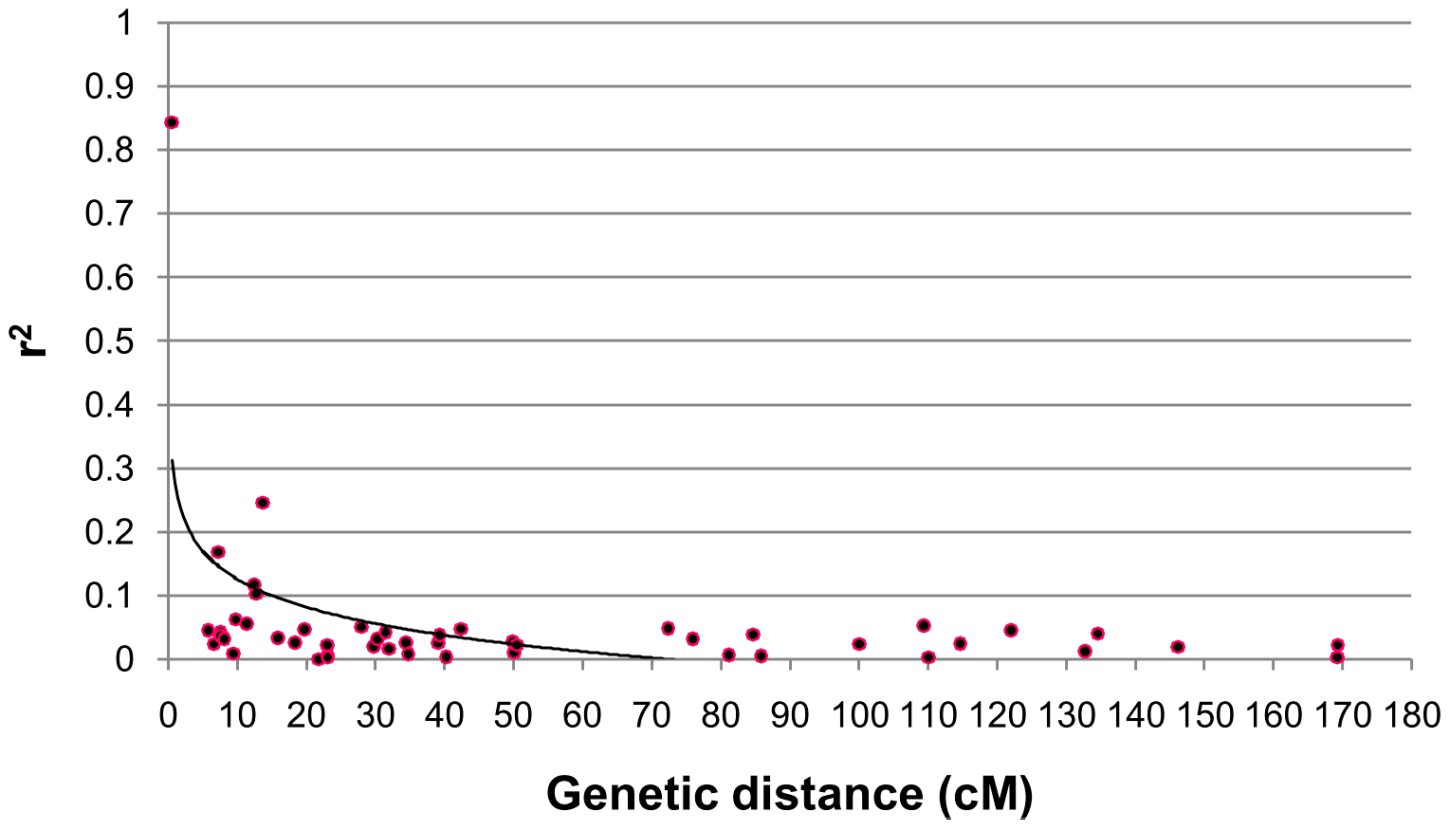
Scatter plots of *r* ^2^ values against genetic distance among linked loci (P≤0.01) in 356 *G. hirsutum* L. accessions. The trend line is a non-linear logarithmic regression curve of *r* ^2^ on genetic distance. LD decayed to the background (*r* ^2^ = 0.1182, P≤0.01), *r* ^2^ = 0.1 and *r* ^2^ = 0.2 level within about 12–13 cM, 17–18 cM and 3–4 cM, respectively.

The *r* ^2^ value and genetic distance of each pair of SSR loci was plotted into a scatter diagram, and then a curve was drawn to describe the trend of LD decay using the nonlinear regression model [43]. The curve exhibited a clear decay of LD with increase in genetic distance (**Figure 2**). In this study, the 99th percentile of *r* ^2^ distribution for unlinked markers, which determined the background level of LD, was 0.1182; and LD decayed to the background level within 12–13 cM. If the threshold of LD decay was set to *r* ^2^ = 0.1 and *r* ^2^ = 0.2, the genome-wide LD extended up to about 17–18 cM and 3–4 cM, respectively (**Figure 2**).

### Variation of phenotypic traits

Seven traits for lint yield and its components were measured in 356 Upland cotton accessions across three different environments. Each trait varied widely (**Table 2**), and the ANOVA showed that the genotype (G) and the interactions between genotype and environmental factors (G×E) were both significant (*P*≤0.01) for all the seven traits. The mean coefficient of variance for FY, SY, BN, BW, LP, LI and SI was 29.09%, 23.19%, 19.36%, 9.11%, 9.33%, 12.48% and 9.02%, respectively, demonstrating that there was a high degree of diversity in lint yield traits of Chinese Upland cotton cultivars. The broad sense heritabilty (*h_B_^2^*) for the seven traits had a range of 27.34–75.77% in the reference population (**Table 2**). The highest *h_B_^2^* value was for LP (75.77%), indicating that LP was less impacted by environmental factors than the other six traits.

**Table 2:** Descriptive statistics, ANOVA and broad-sense heritability (*h_B_^2^*) for yield and its components across three different environments.

Traits^a^	Environments^b^	Mean	SD	Min	Max	CV(%)	G^c^	G×E^d^	*h_B_^2^*(%)
LY	E1	21.66	6.54	8.29	57.91	30.20	**^e^	**	69.10
	E2	27.32	10.24	2.84	68.22	37.48			
	E3	18.72	6.44	6.63	39.27	34.37			
	Mean	22.57	6.57	7.53	42.31	29.09			
SY	E1	54.68	14.02	18.87	133.46	25.65	**	**	55.00
	E2	81.32	25.12	10.88	161.04	30.90			
	E3	52.03	15.46	20.58	93.86	29.72			
	Mean	62.68	14.53	24.23	102.44	23.19			
BN	E1	16.01	3.13	7.00	28.20	19.55	**	**	50.87
	E2	23.06	6.32	4.40	44.30	27.39			
	E3	14.82	3.85	5.70	30.50	26.01			
	Mean	17.96	3.48	8.33	27.73	19.36			
BW	E1	4.58	0.44	3.22	6.04	9.66	**	**	60.96
	E2	4.87	0.59	3.16	6.66	12.03			
	E3	4.85	0.62	2.56	6.48	12.75			
	Mean	4.76	0.43	3.39	6.10	9.11			
LP	E1	39.31	3.57	25.92	50.33	9.08	**	**	75.77
	E2	33.03	3.60	20.03	43.75	10.91			
	E3	35.60	3.59	23.78	46.51	10.07			
	Mean	35.98	3.36	23.76	46.87	9.33			
LI	E1	6.72	0.88	3.74	10.06	13.16	**	**	71.06
	E2	5.92	0.83	3.06	8.70	14.03			
	E3	5.82	0.90	3.00	8.12	15.44			
	Mean	6.15	0.77	3.46	8.17	12.48			
SI	E1	10.35	0.91	8.08	14.71	8.74	**	**	61.09
	E2	12.01	1.28	9.15	15.33	10.67			
	E3	10.51	1.31	6.10	14.35	12.48			
	Mean	10.96	0.99	8.52	13.88	9.02			

^a^ LY: lint yield (g/plant); SY: seed cotton yield (g/plant); BN: bolls per plant; BW: boll weight (g); LP: lint percentage (%); LI: lint index (g/100 seeds); SI: seed index (g/100 seeds).

^b^ E1, E2, and E3 indicate Jiangpu in 2009, Dafeng in 2010 and Zhengzhou in 2010, respectively.

^c^ Genotype across different environments.

^d^ Genotype and environment interaction

^e^ Significant at P<0.01 level.

Phenotypic correlation analysis showed that there were significant positive correlations between lint yield and its most components, while the negative correlation between lint yield and SI was also significant (**Table 3**). The correlation coefficients for LY with SY, BN, BW, LP, LI and SI were 0.963, 0.869, 0.461, 0.704, 0.671, and −0.257, respectively.

**Table 3:** Phenotypic correlations among lint yield and its components based on trait means of 356 upland cotton accessions across three environments.

Traits^a^	LY	SY	BN	BW	LP	LI
SY	0.963***					
BN	0.869***	0.895***				
BW	0.461**	0.497***	0.144			
LP	0.740***	0.544***	0.523***	0.229***		
LI	0.671***	0.541***	0.384***	0.554***	0.796***	
SI	−0.257***	−0.119*	−0.311***	0.424***	−0.498***	0.121*

^a^ See Table 2 for abbreviations.

### Markers associated with lint yield and its components

The marker-trait AM was performed with the MLM model, considering both kinship (K) and population structure (Q), implemented in TASSEL software. At the *α* = 0.01 (−log_10_ *P* = 2) level, a total of 195 significant associations were detected between 82 SSR markers and seven lint yield traits (Table S4). Among these, most of the associations (125 of 195) were detected in only one environment, and the proportion of phenotypic variation explained by markers ranged from 0.0152 to 0.0940, with an average of 0.0370 (Table S4).

In this study, 145 markers were used for detecting association, so the same statistical test was performed 145 times at a significance level of 0.01, and the experimental type I error rate would be much higher than 0.01. To overcome this problem, the Bonferroni correction (*P*≤0.05/145, −log_10_ *P*≥3.46) was used to obtain an appropriate significance threshold [44]. After Bonferroni correction, 55 associations were found to be significant between 26 SSR markers and seven lint yield traits, and the results are shown in **Table 4**. Most (41 associations between 23 SSR markers and seven lint yield traits) of the associations could be detected in more than one environment, and the proportion of phenotypic variation explained by markers ranged from 0.0163 to 0.0940, with an average of 0.0451. The number of SSR markers associated with LY, SY, BN, BW, LP, LI and SI were 9, 4, 6, 4, 14, 17 and 1, respectively. Seventeen loci were co-associated with two or more different traits (**Table 4**). For example, NAU3269 (Chr. 5) and NAU3100 (Chr. 23) were simultaneously associated with FY, SY, BN, LP, and LI, and most of the lint yield-associated loci were associated with at least one of its components.

**Table 4:** SSR marker loci significantly associated with lint yield traits and their explained proportion of phenotypic variation in three different environments.

Traits^a^	Marker loci	Chr.	Position	−Log_10_ *P*			*R^2^*		
				E1^b^	E2^b^	E3^b^	E1^b^	E2^b^	E3^b^
LY	NAU3269^c^	A05(Chr.05)	182.215	3.43	4.42	3.33	0.0283	0.0460	0.0305
	NAU5166^c^	A10(Chr.10)	23.423	ns	5.49	ns		0.0540	
	NAU2935	A10(Chr.10)	51.592	ns	4.00	ns		0.0472	
	JESPR204^c^	A13(Chr.13)	59.714	4.12	3.00	ns	0.0460	0.0408	
	CIR246^c^	D02(Chr.14)	112.473	ns	ns	3.55			0.0444
	BNL3594^c^	D06(Chr.25)	7.66	ns	4.51	ns		0.0765	
	TMK19^c^	D06(Chr.25)	70.38	4.30	2.77	3.97	0.0421	0.0321	0.0428
	NAU3100^c^	D09(Chr.23)	25.262	3.67	5.38	4.31	0.0408	0.0708	0.0541
	NAU2776^c^	D10(Chr.20)	9.452	ns	3.81	2.54		0.0441	0.0276
SY	NAU3269	A05(Chr.05)	182.215	2.25	4.16	2.43	0.0185	0.0441	0.0219
	CIR246	D02(Chr.14)	112.473	ns	ns	4.23			0.0536
	BNL3594	D06(Chr.25)	7.66	ns	4.61	ns		0.0797	
	NAU3100	D09(Chr.23)	25.262	ns	4.03	2.82		0.0561	0.0384
BN	NAU6584	A03(Chr.03)	74.975	3.53	2.29	ns	0.0393	0.0291	
	NAU3269	A05(Chr.05)	182.215	2.08	3.52	3.45	0.0174	0.0364	0.0344
	BNL3594	D06(Chr.25)	7.66	ns	3.62	ns		0.0667	
	TMK19	D06(Chr.25)	70.38	3.58	ns	2.41	0.0371		0.0284
	NAU493	D07(Chr.16)	113.413	3.91	ns	ns	0.0338		
	NAU3100	D09(Chr.23)	25.262	ns	3.48	ns		0.0492	
BW	BNL1414	A09(Chr.09)	95.911	ns	3.26	3.72		0.0418	0.0445
	NAU4047	A12(Chr.12)	11.118	3.29	4.17	ns	0.0332	0.0461	
	NAU3398	A13(Chr.13)	3.311	3.47	3.46	ns	0.0565	0.0603	
	JESPR208	D09(Chr.23)	118.225	ns	4.01	3.69		0.0515	0.0445
LP	NAU3269	A05(Chr.05)	182.215	3.54	2.21	2.72	0.0318	0.0202	0.0259
	NAU5166	A10(Chr.10)	23.423	2.33	4.36	2.74	0.0180	0.0410	0.0244
	NAU2508	A10(Chr.10)	128.028	4.45	3.62	4.68	0.0523	0.0481	0.0609
	NAU980	A11(Chr.11)	0	3.40	ns	3.75	0.0565		0.0672
	JESPR135	A11(Chr.11)	55.787	3.79	6.64	3.80	0.0316	0.0645	0.0354
	NAU3398	A13(Chr.13)	3.311	3.50	ns	ns	0.0499		
	JESPR204	A13(Chr.13)	59.714	3.73	2.96	2.22	0.0441	0.0396	0.0311
	BNL3590	D03(Chr.17)	39.284	4.70	ns	2.89	0.0491		0.0338
	TMK19	D06(Chr.25)	70.38	5.97	4.58	3.67	0.0604	0.0518	0.0419
	NAU3100	D09(Chr.23)	25.262	4.50	3.35	2.89	0.0535	0.0458	0.0400
	NAU3917	D10(Chr.20)	31.125	ns	3.57	ns		0.0403	
	BNL1404	D11(Chr.21)	33.571	4.48	6.20	3.51	0.0381	0.0601	0.0323
	Gh508	D11(Chr.21)	54.48	2.15	3.37	3.81	0.0163	0.0306	0.0354
	NAU2361	D11(Chr.21)	101.215	4.05	4.30	4.03	0.0637	0.0734	0.0703
LI	NAU3269	A05(Chr.05)	182.215	4.98	2.77	2.38	0.0492	0.0263	0.0232
	NAU980	A11(Chr.11)	0	8.85	2.34	2.80	0.0896	0.0476	0.0572
	JESPR135	A11(Chr.11)	55.787	ns	5.43	2.34		0.0520	0.0209
	Gh369	A11(Chr.11)	84.701	3.77	2.09	ns	0.0479	0.0300	
	NAU1151	A12(Chr.12)	97.965	6.08	ns	ns	0.0563		
	NAU3398	A13(Chr.13)	3.311	6.01	2.49	ns	0.0819	0.0415	
	JESPR204	A13(Chr.13)	59.714	ns	3.68	ns		0.0481	
	CIR246	D02(Chr.14)	112.473	4.75	3.14	ns	0.0577	0.0417	
	BNL3590	D03(Chr.17)	39.284	3.30	2.70	3.47	0.0367	0.0313	0.0426
	NAU2233	D05(Chr.19)	171.278	4.86	2.15	ns	0.0596	0.0304	
	TMK19	D06(Chr.25)	70.38	4.83	4.02	ns	0.0519	0.0455	
	NAU3100	D09(Chr.23)	25.262	5.90	2.74	3.47	0.0719	0.0384	0.0493
	NAU2776	D10(Chr.20)	9.452	ns	ns	3.57			0.0430
	NAU3917	D10(Chr.20)	31.125	ns	3.66	2.03		0.0413	0.0245
	BNL1404	D11(Chr.21)	33.571	ns	5.85	2.72		0.0564	0.0250
	Gh508	D11(Chr.21)	54.48	ns	3.67	3.41		0.0338	0.0327
	NAU2361	D11(Chr.21)	101.215	6.27	5.16	4.58	0.0940	0.0845	0.0812
SI	NAU493	D07(Chr.16)	113.413	3.70	2.85	5.24	0.0385	0.0290	0.0575

^a^ See Table 2 for abbreviations.

^b^ E1: Jiangpu in 2009; E2: Dafeng in 2010; E3: Zhengzhou in 2010.

^c^ Markers associated with lint yield simultaneously associated more than one its component.

### Favorable QTL alleles and their transmission in Chinese Upland cotton cultivars

Phenotypic effects of each QTL allele for the 41 associated loci detected in more than one environment were measured according to the method mentioned above, and 5, 2, 3, 4, 12, 14 and 1 favorable alleles for FY, SY, BN, BW, LP, LI and SI were identified, respectively. Phenotypic effects and representative accessions for each favorable allele are shown in **Table 5**. Among the favorable alleles, NAU3100-2 had the most positive phenotypic effect for FY and SY, and increased FY and SY by 3.61 g and 7.27 g, respectively; NAU6584-2, NAU3398-2, NAU5166-2 and NAU3917-2 increased BN, BW, LP and LI by 0.89, 0.42 g, 4.93% and 0.94 g, respectively; while NAU493-1 deceased SI by 0.17 g.

**Table 5:** Favorable QTL alleles, their phenotypic effects (*a_i_*) and representative accessions.

Traits^a^	Favorable allele	*a_i_*	Accessions	Representative accessions^b^
LY	NAU3269-2	0.27	133	Simian3, Zhongmiansuo9, Huakangmian1
	JESPR204-1	0.70	314	Simian3, Zhongmiansuo9, P164-2
	TMK19-2	1.02	240	Simian3, Zhongmiansuo9, P164-2
	NAU3100-2	3.61	87	Simian3, Zhongmiansuo9, Lumianyan16
	NAU2776-1	0.85	151	Zhongmiansuo9, P164-2, Lumianyan16
SY	NAU3269-2	0.42	133	Zhongmiansuo9, Zhongmiansuo19, Simian3
	NAU3100-2	7.27	87	Zhongmiansuo9, Han4849, Lumianyan16
BN	NAU6584-2	0.89	217	Lumianyan16, Zhongmiansuo44, Zhongmiansuo9
	NAU3269-2	0.08	133	Zhongmiansuo9, Wanmian73-10, Simian3
	TMK19-2	0.45	235	Zhongmiansuo44, Zhongmiansuo9, Wanmian17
BW	BNL1414-2	0.18	93	Zhongmiansuo18, Zhongmiansuo5, I40005
	NAU4047-2	0.03	221	Zhongmiansuo18, Zhongmiansuo5, I40005
	NAU3398-2	0.42	22	Zhongmiansuo5, I40005, Hua101
	JESPR208-2	0.20	86	Zhongmiansuo18, Zhongmiansuo5, I40005
LP	NAU3269-2	0.23	133	Simian3, Ekangmian9, Huakangmian1
	NAU5166-2	4.93	8	Simian3, Huakangmian1, Sumian4
	NAU2508-2	0.36	113	Nannongzao, 86-1, Yu668
	NAU980-3	2.79	9	Ekangmian6, Emian16, Ekangmian10
	JESPR135-1	0.13	343	XiangSC-24, Simian3, Ekangmian9
	JESPR204-1	0.36	309	XiangSC-24, Simian3, Ekangmian9
	BNL3590-1	0.26	327	XiangSC-24, Simian3, Ekangmian9
	TMK19-2	0.58	235	XiangSC-24, Simian3, Huakangmian1
	NAU3100-2	1.55	86	Simian3, Ekangmian9, Nannongzao
	BNL1404-1	0.13	343	XiangSC-24, Simian3, Ekangmian9
	Gh508-1	0.07	347	XiangSC-24, Simian3, Ekangmian9
	NAU2361-3	0.75	73	Ekangmian9, Yu668, Yumian21
LI	NAU3269-2	0.01	133	Huakangmian1, I40005, Ekangmian9
	NAU980-3	0.84	9	I40005, Zhongmiansuo5, Hua101
	JESPR135-1	0.02	343	Huakangmian1, Emian23, I40005
	Gh369-3	0.11	8	Emian16, Ekangmian8, Yumian20
	NAU3398-2	0.80	22	Huakangmian1, I40005, Zhongmiansuo5
	CIR246-3	0.63	16	Hua101, Zhongmiansuo41, Yumian9
	BNL3590-1	0.06	327	Huakangmian1, Emian23, I40005
	NAU2233-1	0.01	212	Huakangmian1, Emian23, I40005
	TMK19-2	0.10	235	Huakangmian1, Emian23, I40005
	NAU3100-2	0.38	86	I40005, Zhongmiansuo5, Hua101
	NAU3917-2	0.94	6	Huakangmian1, Simian4, Sumian9
	BNL1404-1	0.03	343	Huakangmian1, Emian23, I40005
	Gh508-1	0.01	347	Huakangmian1, Emian23, I40005
	NAU2361-3	0.28	73	Emian23, I40005, Zhongmiansuo5
SI	NAU493-1	−0.17	230	Chaoyangmian1, Xuzhou1818, XiangSC-24

^a^ See Table 2 for abbreviations.

^b^ Representative accessions are the top-3 entries for the target trait value of accessions with the corresponding favorable allele.

Allele frequencies of the 23 favorable alleles in the CK group and the six Chinese historically released cultivar groups are summarized in **Table 6**. Based on allele frequencies across the different groups, these favorable alleles could be categorized into three classes. The alleles in the first class, such as JESPR135-1, BNL1404-1 and Gh508-1, presented in the founder cultivars and with high frequency in all populations, might have been passed down stably from the original parents and were almost fixed in modern cultivars by selection. Alleles in the second class, such as BNL3269-2, BNL1414-2, NAU3100-2 and JESPR208-2, presented in the founder cultivars and with moderate to low frequency in most populations, should have been underutilized in modern breeding programs. Those in the third class, such as NAU5166-2, NAU980-3, Gh369-3 and CIR246-3, not presented in the founder cultivars and presented at low frequency in modern cultivars, might be from other original parents or could have been generated by mutations and/or recombinations. Favorable alleles, especially of the latter two classes, should have a great potential in future Upland cotton genetic improvement.

**Table 6:** Allele frequency for each favorable QTL allele in historically released Chinese Upland cotton cultivar groups.^a^

Favorable alleles	CK	I	II	III	IV	V	VI	Total
NAU6584-2	0.3333	0.5000	0.6923	0.7692	0.6747	0.4720	0.7755	0.6096
NAU3269-2	0.6667	0.5769	0.5385	0.6154	0.3976	0.3040	0.1020	0.3736
BNL1414-2	0.5000	0.3077	0.1923	0.4872	0.2410	0.2320	0.1633	0.2612
NAU5166-2	0.0000	0.0000	0.0000	0.0500	0.0000	0.0320	0.0417	0.0225
NAU2508-2	0.4000	0.1200	0.2400	0.2250	0.3012	0.3952	0.4694	0.3333
NAU980-3	0.0000	0.0200	0.0000	0.0256	0.0244	0.0369	0.0000	0.0233
JESPR135-1	1.0000	0.8462	1.0000	1.0000	0.9759	0.9840	1.0000	0.9775
Gh369-3	0.0000	0.0000	0.0000	0.0000	0.0241	0.0480	0.0000	0.0225
NAU4047-2	0.6667	0.5000	0.5769	0.6154	0.5663	0.6400	0.7551	0.6208
NAU3398-2	0.0000	0.0385	0.0000	0.1026	0.0843	0.0720	0.0204	0.0618
JESPR204-1	1.0000	0.8462	0.8077	0.7692	0.8675	0.9200	0.9388	0.8820
CIR246-3	0.0000	0.0000	0.0000	0.0256	0.0241	0.0800	0.0612	0.0449
BNL3590-1	0.5000	0.8077	0.8462	0.8462	0.9036	0.9440	0.8776	0.8904
NAU2233-1	0.6667	0.4231	0.5000	0.3590	0.5542	0.6560	0.8163	0.5955
TMK19-2	0.6667	0.5000	0.5769	0.6667	0.6024	0.7280	0.8163	0.6742
NAU493-1	0.6667	0.5000	0.7308	0.6923	0.6265	0.6800	0.5714	0.6461
NAU3100-2	0.3333	0.2308	0.0769	0.2308	0.2289	0.2400	0.3673	0.2444
JESPR208-2	0.5000	0.3077	0.1154	0.4359	0.2289	0.2240	0.1633	0.2444
NAU2776-1	0.1667	0.4231	0.3462	0.5385	0.4458	0.4480	0.3061	0.4242
NAU3917-2	0.0000	0.0000	0.0000	0.0513	0.0000	0.0240	0.0204	0.0169
BNL1404-1	1.0000	0.8462	1.0000	1.0000	0.9759	0.9840	1.0000	0.9775
Gh508-1	1.0000	0.9231	1.0000	0.9750	1.0000	0.9920	1.0000	0.9888
NAU2361-3	0.2143	0.0769	0.0417	0.1538	0.1125	0.2810	0.3673	0.2066

^a^ CK, I, II, III, IV, V and VI indicates the founder parent group (CK), and the Chinese cultivars released in 1930–1960, 1961–1970, 1971–1980, 1981–1990, 1991–2000 and 2000–2005, respectively.

## Discussion

### Genetic diversity and population structure of the association panel

A suitable association mapping panel should embrace as much phenotypic and genotypic diversity as can be reliably measured in common environments [45]. Most Upland cotton cultivars developed in China were derived from a few germplasm resources introduced from abroad and therefore the genetic base is narrow [46]–[47]. It is especially critical to select samples that encompass genetic diversity as much as possible. In this study, the 356 Upland cotton accessions, which can normally flower and ripen for target trait evaluation, were chosen from more than 1000 cultivars and breeding lines in CRI-CAAS and NAU germplasm collections. The phenotypic measurments in three different locations indicated that there was a high degree of diversity in lint yield and its component traits (**Table 2**). Of the 381 SSR markers, only 145 were found to be polymorphic in the 356 Upland cotton accessions, indicating that intraspecific genetic diversity is far less than interspecific diversity; for the reference linkage map of allotetraploid cotton was constructed with a BC_1_ mapping population derived from an interspecific cross (*G. hirsutum* TM-1×*G. barbadense* Hai7124) [33]. Eighty percent of the 145 polymorphic loci only generated two or three alleles, and the allele frequencies of 131 of the 415 alleles were <0.05 (Table S2), showing that the genetic diversity in this panel is relatively low, which might affect the QTL detection power of AM in Upland cotton.

Many crops have a long and complex history of domestication and breeding, such as Upland cotton, and complex population structures may confound AM [48]. It is important to consider the influence of population structure and relationships between individuals in the AM panel [27]–[28]. The model-based evaluation of the population structure of the 356 Upland cotton cultivars showed that the population could be divided into two major subpopulations (**Figure 1**). Of the 115 accessions in P1 group, 63, 46 and 6 cultivars were from the Yellow River, North/Northwest China and Yangtze River cotton growing regions, respectively. Out of the 241 accessions in P2 group, 116 were from Yellow River, 107 from Yangtze River, 10 from North/Northwest China cotton growing regions and eight from abroad (Table S1). The North/Northwest China, Yellow River and Yangtze River region represented short-, middle- and long-growth-period cotton cultivation area in China, respectively. The P1 group contained almost all cultivars with early maturity and part of cultivars with middle maturity, while the P2 group contained almost all cultivars with late maturity and part of cultivars with middle maturity [32].

### Linkage disequilibrium in Upland cotton

The extent of LD can provide information for the needed marker density and mapping resolution in AM study [25]. LD decay had been repeatedly estimated in many plant species [26],[48], while that was limited in cotton. Abdurakhmonov et al. (2008) performed a pioneer estimation [20]. They reported that, in a panel contained 285 exotic *Gossypium hirsutum* accessions, the genome-wide LD (*r* ^2^≥0.1) declined at <10 cM in the landrace stocks and >30 cM in variety germplasm, but at r^2^≥0.2 which reduced to about 1–2 cM and 6–8 cM, respectively. In another panel composed of 335 *G. hirsutum* variety germplasm, the genome-wide LD extended up to 25 cM at *r* ^2^≥0.1 and reduced to about 5–6 cM at *r* ^2^≥0.2 [21]. In the present panel, the average *r* ^2^ of locus pairs was 0.0103, and 18.29% were significant (P≤0.01) (**Table 1**), which is higher than that (13% siganificant at P≤0.01) reported by Abdurakhmonov et al. [21]. In our panel, the LD decayed to genome background level (*r* ^2^ = 0.1182) within 12–13 cM (Fig. 2). If the threshold of LD decay was set to *r* ^2^ = 0.1 and *r* ^2^ = 0.2, the genome-wide LD extended up to 17–18 cM and 3–4 cM (**Figure 2**), respectively, which is shorter than those previously reported [20]–[21].

In the entire panel and subpopulations, both average *r* ^2^ and proportion of significant LD for linked loci were all higher than those for unlinked markers (**Table 1**), demonstrating that physical linkage is predominant in determining LD compared with random forces in the present association panel [48]. Therefore this Upland cotton panel is suitable for association analysis and has the potential to identify QTLs in an interval equivalent to the distance of LD decay of 3–4 cM. Based on the LD decay in the panel of 335 *G. hirsutum* varieties, it is suggested that about 1,000 polymorphic markers be required for successful association mapping with LD extending to 5–6 cM [21]. In our panel, the LD decayed faster, suggesting that more markers are probably needed for genome wide association analysis (GWAS) of complex traits. As is often the case in self-pollinated crops [26], the level of LD in the Upland cotton genome was moderately high, suggesting that the mapping resolution gained from LD is likely to be limited. Given that genomic selection is less challenging than map-based cloning, the level of LD in the present population would guarantee that the identified SSR markers would facilitate breeding for high-yield in Upland cotton.

### QTLs for lint yield identified by association mapping

Association mapping can be affected by many factors, such as population structure, relatedness among accessions, small sample size, and low frequency of specific alleles; these may increase the detection of false positive associations [25],[28]. In this study, the AM was performed in a moderately large sized panel (356 accessions) with the optimal model of MLM, considering both population structure and relatedness, to detect SSR markers associated with lint yield and its components. A total of 195 significant associations were detected between 86 SSR markers and 7 lint yield and yield component traits at the *α* = 0.01 (−log_10_ *P* = 2) level (Table S4). It is very difficult to say which significance level is acceptable in a given association study. The use of stringent probability thresholds will reduce the danger of false positives, but meanwhile has the risk of rejecting true positives caused by setting the thresholds too high [49]. Since the present study aimed at mining favorable alleles of main QTL for lint yield, a relatively stringent significance threshold (*P*≤0.05/145, −log_10_ *P*≥3.46) for the Bonferroni correction was adopted to reduce the experimental type I error rate induced by multiple tests [44]. After Bonferroni correction (*P*≤0.05/145, −log_10_ *P*≥3.46), 55 associations remained significant and 74.55% (41 of 55) could be detected in more than one environment (**Table 4**). Population size had been considered as a factor that severely affects the QTL detection power in AM [18],[26]. Many more associations were detected in our present panel than in another 81-accession panel between the same markers and target traits at the same significance level [24, unpublished data].

Although the markers used in different studies are different, and QTL mapping results are not easy to be compared, some of the marker associations detected in this study were consistent with QTLs for lint yield and its components that had been mapped previously by conventional linkage mapping. The locus JESPR204 associated with LY on chromosome 13 (detected in 2 environments) was located in the same region as a QTL identified by Wu et al. [50]; TMK19 (Chr. 25, detected in 3 environments) was consistent with the results of our prevenient study [51]; NAU3100 (Chr. 23, detected in 2 environments) associated with SY was consistent with that found in the study of Wang et al. [8]; NAU980 (Chr. 11, detected in 2 environments), BNL3590 (Chr. 17, detected in 2 environments) and TMK19 (Chr. 25, detected in 3 environments) associated with LP were consistent with several earlier reports [11],[51],[52]; CIR246 (Chr. 14, detected in 2 environments) and NAU3100 (Chr. 23, detected in 3 environments) associated with LI were also consistent with results from several recent QTL mapping studies [11],[51],[52].

Moreover, in our study, seventeen markers were co-associated with two or more different traits, and most of the lint yield-associated markers were associated with at least one of its components, which coincided with phenotypic correlations among these traits. This could result from pleiotropy of a single causal gene or tight linkage of multiple causal genes. We found that 10 of 14 markers associated with LP were detected in all three environments, which was consistent with the phenotypic statistical analysis that LP possessed the highest broad-sense heritability (*h*_B_^2^ = 75.77%). The phenotype of complex traits often results from the combined actions of multiple genes and environmental factors, all these can easily lead to lost heritability [18]; only those traits with high heritability can be stably detected. The resulting stably associated markers should be useful for cotton breeding with broad adaptability to different environments.

### Favorable alleles and their potential application in future cotton breeding programs

Since most Upland cotton cultivars developed in China were derived from limited founder parents, there is great challenge in genetic improvement and high risk of vulnerability to changing climate. New variations that have emerged and accumulated during the long breeding history in China should be fully exploited and additional diversity should be introduced into breeding programs to broaden the genetic basis of Chinese Upland cotton. By comparing the average phenotypic value of each allele for target traits in the 41 stably detected associations, we identified 5, 2, 3, 4, 12, 14 and 1 favorable alleles for FY, SY, BN, BW, LP, LI and SI, respectively (**Table 5**). We suggest that a multi-parent population should be constructed using cultivars that possess most of the favorable alleles, and in the meantime, a ranking system for MAS or genomic selection should be developed based on the results of AM. Favorable alleles that were passed down from the founder parents and have been almost fixed in modern cultivars formed the basis of lint yield of Chinese Upland cotton, and should be treated as fundamental elements in order to reject deleterious alleles at the corresponding loci. Alleles either absent in the founder cultivars or present at moderate to low frequencies in most cultivar groups have been underutilized in modern breeding programs, and should be regarded as essential elements for increasing lint yield potential.

Lint yield of cotton is the result of series components and their interactions, such as boll number, boll weight, lint percentage, lint index, and seed index. Developing potentially high-yielding cultivars thus relies to some extent on selecting the appropriate yield components. As some of the QTLs were associated with more than one yield component, favorable alleles must be treated with caution. Positively co-associated genetic loci could simultaneously improve multiple target traits, while negative linkages must be broken. In summary, the favorable alleles indentified in this study have great potential for developing high-yielding Upland cotton cultivars in future breeding programs.

## Supporting Information

Table S1 Detailed information of the 356 accessions in the association panel.
(DOC)

Table S2 Detailed information of 145 polymorphic SSR markers.
(DOC)

Table S3 Genetic diversity in different historically released cultivar groups.
(DOC)

Table S4 SSR markers significantly (*P*≤0.01, −log_10_ *P*≥2.0) associated with lint yield traits and their explained phenotypic variation across three different environments.
(DOC)
