# Supplementary material for: Favorable QTL Alleles for Yield and Its Components Identified by Association Mapping in Chinese Upland Cotton Cultivars
Source: PLoS One. 2013 Dec 26;8(12):e82193. doi: 10.1371/journal.pone.0082193 (PMC3873261; doi:10.1371/journal.pone.0082193)
Supplement: Table S1 — Detailed information of the 356 accessions in the association panel. (DOC) [file pone.0082193.s001.doc]

**Table S1** Detailed information of the 356 accessions in the association panel

| **Code** | **Cultivars** | **Growing region** | **Released year** | **Subpopulation^a^** |
| --- | --- | --- | --- | --- |
| 1 | Guannong1 | North China | 1930 | P1 |
| 2 | Lixian72 | Yangtze River | 1937 | P2 |
| 3 | Jingsimian | Huang River | 1942 | P1 |
| 4 | 52-128 | Yangtze River | 1952 | P2 |
| 5 | Yishuhong | Yangtze River | 1953 | P2 |
| 6 | Qianmian465 | Yangtze River | 1954 | P1 |
| 7 | Xuzhou209 | Huang River | 1955 | P2 |
| 8 | Jinyu5 | North China | 1955 | P1 |
| 9 | Dongting1 | Yangtze River | 1955 | P2 |
| 10 | Chuanjian1 | Yangtze River | 1955 | P2 |
| 11 | Chaoyangmian1 | North China | 1956 | P1 |
| 12 | Huabei103 | Huang River | 1957 | P1 |
| 13 | Shanmian1 | Huang River | 1957 | P1 |
| 14 | Yapengmian | Yangtze River | 1957 | P1 |
| 15 | Huabei21 | Huang River | 1958 | P2 |
| 16 | Huabei113 | Huang River | 1958 | P1 |
| 17 | jinmian1 | North China | 1958 | P2 |
| 18 | Chuanjian3 | Yangtze River | 1958 | P2 |
| 19 | Zhongmiansuo2 | Huang River | 1959 | P2 |
| 20 | Liaomian1 | North China | 1959 | P1 |
| 21 | Shiduan5 | Huang River | 1960 | P2 |
| 22 | Zhongmiansuo3 | Huang River | 1960 | P2 |
| 23 | Zhong99 | Huang River | 1960 | P2 |
| 24 | Gaomi933 | Huang River | 1960 | P2 |
| 25 | Jingzhong200 | Huang River | 1960 | P2 |
| 26 | Zhemian3 | Yangtze River | 1960 | P2 |
| 27 | Xuzhou1818 | Huang River | 1961 | P2 |
| 28 | Emian1 | Yangtze River | 1962 | P2 |
| 29 | Xiangmian1 | Yangtze River | 1962 | P2 |
| 30 | 57-681 | Yangtze River | 1963 | P2 |
| 31 | Emian2 | Yangtze River | 1963 | P2 |
| 32 | Xiangmian2 | Yangtze River | 1963 | P2 |
| 33 | Chaoyangmian2 | North China | 1964 | P1 |
| 34 | Emian3 | Yangtze River | 1964 | P2 |
| 35 | Shan401 | Huang River | 1965 | P2 |
| 36 | Emian4 | Yangtze River | 1965 | P2 |
| 37 | Shanmian3 | Huang River | 1966 | P2 |
| 38 | Nantong5 | Yangtze River | 1966 | P2 |
| 39 | Emian5 | Yangtze River | 1966 | P2 |
| 40 | Shangqiu24 | Huang River | 1967 | P2 |
| 41 | Zhong8010 | Huang River | 1968 | P2 |
| 42 | Jingzhong169 | Huang River | 1968 | P1 |
| 43 | Jinmian3 | North China | 1968 | P1 |
| 44 | Jinmian2 | North China | 1968 | P1 |
| 45 | Jinmian4 | North China | 1968 | P1 |
| 46 | Jinmian5 | North China | 1968 | P1 |
| 47 | Keyi2 | Huang River | 1970 | P1 |
| 48 | Jinhan3号 | Huang River | 1970 | P2 |
| 49 | Henan69 | Huang River | 1970 | P2 |
| 50 | Jiangsumian1 | Yangtze River | 1970 | P2 |
| 51 | Jing'an508 | Yangtze River | 1970 | P2 |
| 52 | Emian6 | Yangtze River | 1970 | P2 |
| 53 | Jimian1 | Huang River | 1971 | P2 |
| 54 | Zhongmiansuo5 | Huang River | 1971 | P2 |
| 55 | Liaomian3 | North China | 1971 | P2 |
| 56 | Jiangsumian3 | Yangtze River | 1971 | P2 |
| 57 | Emian7 | Yangtze River | 1971 | P2 |
| 58 | Shanmian4 | Huang River | 1972 | P2 |
| 59 | Keyi181 | Huang River | 1972 | P1 |
| 60 | Xuzhou58 | Huang River | 1972 | P1 |
| 61 | Liaomian4 | North China | 1972 | P1 |
| 62 | Wanmian1 | Yangtze River | 1972 | P2 |
| 63 | Emian9 | Yangtze River | 1972 | P2 |
| 64 | Zhongmiansuo9 | Huang River | 1973 | P2 |
| 65 | Hemian3 | Huang River | 1973 | P2 |
| 66 | Xuzhou142 | Huang River | 1973 | P2 |
| 67 | Shan3619 | Huang River | 1973 | P2 |
| 68 | Simian1 | Yangtze River | 1973 | P2 |
| 69 | Gangmian1 | Yangtze River | 1973 | P2 |
| 70 | Shanmian5 | Huang River | 1974 | P2 |
| 71 | Heishanmian1 | North China | 1974 | P1 |
| 72 | Humian204 | Yangtze River | 1974 | P2 |
| 73 | Nantong12 | Yangtze River | 1974 | P2 |
| 74 | Jimian2 | Huang River | 1975 | P2 |
| 75 | Shanmian6 | Huang River | 1975 | P2 |
| 76 | Jimian6 | Huang River | 1975 | P1 |
| 77 | 86-1 | Huang River | 1975 | P2 |
| 78 | Emian10 | Yangtze River | 1975 | P2 |
| 79 | Shan5245 | Huang River | 1976 | P2 |
| 80 | Shanmian9 | Huang River | 1976 | P2 |
| 81 | Henan79 | Huang River | 1976 | P2 |
| 82 | Wanmian2 | Yangtze River | 1977 | P2 |
| 83 | Gangmian2 | Yangtze River | 1977 | P2 |
| 84 | Yukang1 | Huang River | 1978 | P1 |
| 85 | Jimian3 | Huang River | 1978 | P2 |
| 86 | Shan1155 | Huang River | 1978 | P2 |
| 87 | Wanmian3 | Yangtze River | 1978 | P2 |
| 88 | Annong121 | Yangtze River | 1978 | P2 |
| 89 | Lumian1 | Huang River | 1979 | P2 |
| 90 | Liaomian6 | North China | 1980 | P2 |
| 91 | Emian11 | Yangtze River | 1980 | P2 |
| 92 | Yumian1 | Huang River | 1981 | P2 |
| 93 | Yu79-10 | Huang River | 1981 | P2 |
| 94 | Jimian13 | Huang River | 1981 | P2 |
| 95 | Jinmian4 | Huang River | 1981 | P1 |
| 96 | Jimian7 | Huang River | 1982 | P2 |
| 97 | Jinmian5 | Huang River | 1982 | P1 |
| 98 | Xumian6 | Huang River | 1982 | P2 |
| 99 | Xiangmian10 | Yangtze River | 1982 | P2 |
| 100 | Siyang331 | Yangtze River | 1982 | P2 |
| 101 | Lumian4 | Huang River | 1983 | P2 |
| 102 | Jinmian7 | Huang River | 1983 | P1 |
| 103 | Jinmian6 | Huang River | 1983 | P1 |
| 104 | Jimian8 | Huang River | 1983 | P2 |
| 105 | Lumian2 | Huang River | 1983 | P2 |
| 106 | Lumian3 | Huang River | 1983 | P2 |
| 107 | Lumian5 | Huang River | 1983 | P2 |
| 108 | 86-6 | Huang River | 1983 | P2 |
| 109 | Cangzhou7315-38 | Huang River | 1983 | P1 |
| 110 | Jinmian8 | Huang River | 1983 | P2 |
| 111 | Liaomian7 | North China | 1983 | P1 |
| 112 | Liaomian8 | North China | 1983 | P1 |
| 113 | Beinong1 | Huang River | 1984 | P2 |
| 114 | Jimian10 | Huang River | 1984 | P2 |
| 115 | Lumian6 | Huang River | 1984 | P2 |
| 116 | Shan6192 | Huang River | 1984 | P2 |
| 117 | Liaomian9 | North China | 1984 | P1 |
| 118 | Daihongdai | Yangtze River | 1984 | P2 |
| 119 | Simian2 | Yangtze River | 1984 | P2 |
| 120 | Emian12 | Yangtze River | 1984 | P2 |
| 121 | Xiang4108 | Yangtze River | 1984 | P2 |
| 122 | Xiangmian11 | Yangtze River | 1985 | P2 |
| 123 | Eguangmian | Yangtze River | 1985 | P2 |
| 124 | Chuan73-27 | Yangtze River | 1985 | P2 |
| 125 | Sumian2 | Yangtze River | 1985 | P2 |
| 126 | Wanmian73-10 | Yangtze River | 1985 | P2 |
| 127 | Jimian11 | Huang River | 1986 | P2 |
| 128 | Jimian12 | Huang River | 1986 | P2 |
| 129 | Yuwu19 | Huang River | 1986 | P2 |
| 130 | Chuan414 | Yangtze River | 1986 | P2 |
| 131 | Yumian2 | Huang River | 1987 | P2 |
| 132 | Xinluzao3 | North-west China | 1987 | P1 |
| 133 | Yanmian1 | Yangtze River | 1987 | P2 |
| 134 | 86-4 | Huang River | 1988 | P1 |
| 135 | Yumian3 | Huang River | 1988 | P2 |
| 136 | Jimian14 | Huang River | 1988 | P2 |
| 137 | Jimian15 | Huang River | 1988 | P2 |
| 138 | Jinmian9 | Huang River | 1988 | P1 |
| 139 | Xinluzao2 | North-west China | 1988 | P1 |
| 140 | Xinluzhong2 | North-west China | 1988 | P1 |
| 141 | Xiangmian12 | Yangtze River | 1988 | P2 |
| 142 | Sumian1 | Yangtze River | 1988 | P2 |
| 143 | Yumian5 | Huang River | 1989 | P2 |
| 144 | Zhongmiansuo14 | Huang River | 1989 | P1 |
| 145 | Jinmian10 | Huang River | 1989 | P1 |
| 146 | Zhongmiansuo12 | Huang River | 1989 | P2 |
| 147 | Zhongmiansuo15 | Huang River | 1989 | P2 |
| 148 | Yumian4 | Huang River | 1989 | P2 |
| 149 | Xinluzhong3 | North-west China | 1989 | P2 |
| 150 | Xiangmian13 | Yangtze River | 1989 | P2 |
| 151 | Qingli514 | Huang River | 1990 | P1 |
| 152 | Zhongmiansuo17 | Huang River | 1990 | P2 |
| 153 | Lumian9 | Huang River | 1990 | P2 |
| 154 | Zhongmiansuo16 | Huang River | 1990 | P1 |
| 155 | Zhongmiansuo10 | Huang River | 1990 | P1 |
| 156 | Lumian10 | Huang River | 1990 | P2 |
| 157 | Luwu401 | Huang River | 1990 | P1 |
| 158 | Shanzao2786 | Huang River | 1990 | P2 |
| 159 | Zhongmiansuo18 | Huang River | 1990 | P1 |
| 160 | Xingmian2 | Huang River | 1990 | P2 |
| 161 | Zhongmiansuo11 | Huang River | 1990 | P2 |
| 162 | Jinmian11 | Huang River | 1990 | P1 |
| 163 | Liaomian10 | North China | 1990 | P1 |
| 164 | Esha28 | Yangtze River | 1990 | P2 |
| 165 | Sumian3 | Yangtze River | 1990 | P2 |
| 166 | Zhemian9 | Yangtze River | 1990 | P2 |
| 167 | Ejing92 | Yangtze River | 1990 | P2 |
| 168 | Emian13 | Yangtze River | 1990 | P1 |
| 169 | Emian16 | Yangtze River | 1990 | P2 |
| 170 | Ekangmian1 | Yangtze River | 1990 | P2 |
| 171 | Emian14 | Yangtze River | 1990 | P2 |
| 172 | Yanmian48 | Yangtze River | 1990 | P2 |
| 173 | Xuzhou514 | Yangtze River | 1990 | P2 |
| 174 | I40005 | Yangtze River | 1990 | P2 |
| 175 | Zhongmiansuo13 | Huang River | 1991 | P2 |
| 176 | Yun1729 | Huang River | 1991 | P2 |
| 177 | Yumian6 | Huang River | 1991 | P2 |
| 178 | Xinluzao1 | North-west China | 1991 | P1 |
| 179 | Wanmian5 | Yangtze River | 1991 | P2 |
| 180 | Ganmian8 | Yangtze River | 1991 | P2 |
| 181 | Zhongmiansuo19 | Huang River | 1992 | P1 |
| 182 | Yumian7 | Huang River | 1992 | P1 |
| 183 | Lumian11 | Huang River | 1992 | P2 |
| 184 | Chuanmian56 | Yangtze River | 1992 | P2 |
| 185 | Hua101 | Yangtze River | 1992 | P2 |
| 186 | Sumian4 | Yangtze River | 1992 | P2 |
| 187 | Emian17 | Yangtze River | 1992 | P2 |
| 188 | Simian4 | Yangtze River | 1992 | P2 |
| 189 | Ejing1 | Yangtze River | 1992 | P2 |
| 190 | Jinmian12 | Huang River | 1993 | P2 |
| 191 | Yumian8 | Huang River | 1993 | P2 |
| 192 | Sumian6 | Yangtze River | 1993 | P2 |
| 193 | Ekangmian2 | Yangtze River | 1993 | P2 |
| 194 | Simian3 | Yangtze River | 1993 | P2 |
| 195 | Sumian5 | Yangtze River | 1993 | P2 |
| 196 | Emian19 | Yangtze River | 1993 | P1 |
| 197 | Emian18 | Yangtze River | 1993 | P2 |
| 198 | Zhongmiansuo22 | Huang River | 1994 | P2 |
| 199 | Jimian19 | Huang River | 1994 | P1 |
| 200 | Yumian9 | Huang River | 1994 | P1 |
| 201 | Jinmian16 | Huang River | 1994 | P1 |
| 202 | Zhongmiansuo20 | Huang River | 1994 | P1 |
| 203 | Lu742 | Huang River | 1994 | P1 |
| 204 | Zhongmiansuo21 | Huang River | 1994 | P2 |
| 205 | Yumian11 | Huang River | 1994 | P2 |
| 206 | Liaomian12 | North China | 1994 | P1 |
| 207 | Liaomian13 | North China | 1994 | P1 |
| 208 | Xinluzao5 | North-west China | 1994 | P1 |
| 209 | Xinluzao4 | North-west China | 1994 | P1 |
| 210 | Xinluzhong5 | North-west China | 1994 | P2 |
| 211 | Xiangmian16 | Yangtze River | 1994 | P2 |
| 212 | Sumian7 | Yangtze River | 1994 | P2 |
| 213 | Wanmian8 | Yangtze River | 1994 | P2 |
| 214 | Emian20 | Yangtze River | 1994 | P2 |
| 215 | Zhongmiansuo25 | Huang River | 1995 | P2 |
| 216 | Jinmian19 | Huang River | 1995 | P2 |
| 217 | Zhongmiansuo33 | Huang River | 1995 | P1 |
| 218 | Jinmian18 | Huang River | 1995 | P1 |
| 219 | Jinmian17 | Huang River | 1995 | P1 |
| 220 | Zhongmiansuo23 | Huang River | 1995 | P2 |
| 221 | Sumian9 | Yangtze River | 1995 | P2 |
| 222 | Changkangmain | Yangtze River | 1995 | P1 |
| 223 | Ekangmian3 | Yangtze River | 1995 | P2 |
| 224 | Chuanmian30 | Yangtze River | 1995 | P2 |
| 225 | Sumian8 | Yangtze River | 1995 | P2 |
| 226 | Sumian10 | Yangtze River | 1995 | P2 |
| 227 | Yumian14 | Huang River | 1996 | P1 |
| 228 | Jimian21 | Huang River | 1996 | P1 |
| 229 | Jimian20 | Huang River | 1996 | P2 |
| 230 | Jinmian20 | Huang River | 1996 | P2 |
| 231 | Qinyuan4 | Huang River | 1996 | P2 |
| 232 | Liaomian14 | North China | 1996 | P1 |
| 233 | Liaomian15 | North China | 1996 | P1 |
| 234 | Jinmian6 | North China | 1996 | P1 |
| 235 | Chuanmian243 | Yangtze River | 1996 | P2 |
| 236 | Emian21 | Yangtze River | 1996 | P2 |
| 237 | Zhongmiansuo4133 | Huang River | 1996 | P2 |
| 238 | Jinmian21 | Huang River | 1997 | P1 |
| 239 | Jinmian23 | Huang River | 1997 | P1 |
| 240 | Yumian15 | Huang River | 1997 | P2 |
| 241 | Yumian16 | Huang River | 1997 | P2 |
| 242 | Zhongmiansuo24 | Huang River | 1997 | P1 |
| 243 | Yumian17 | Huang River | 1997 | P2 |
| 244 | Xinluzao8 | North-west China | 1997 | P1 |
| 245 | Xinluzao6 | North-west China | 1997 | P1 |
| 246 | Xinluzao7 | North-west China | 1997 | P1 |
| 247 | Sumian11 | Yangtze River | 1997 | P2 |
| 248 | Zhemian11 | Yangtze River | 1997 | P2 |
| 249 | Ekangmian6 | Yangtze River | 1997 | P2 |
| 250 | Sumian12 | Yangtze River | 1997 | P2 |
| 251 | Zhongmiansuo34 | Huang River | 1998 | P2 |
| 252 | Zhongmiansuo32 | Huang River | 1998 | P1 |
| 253 | Nongda94-7 | Huang River | 1998 | P2 |
| 254 | Jinmian26 | Huang River | 1998 | P1 |
| 255 | Zhongmiansuo27 | Huang River | 1998 | P1 |
| 256 | Zhongmiansuo30 | Huang River | 1998 | P1 |
| 257 | Zhongmiansuo31 | Huang River | 1998 | P1 |
| 258 | Dexiamian1 | Huang River | 1998 | P1 |
| 259 | Jinmian24 | Huang River | 1998 | P1 |
| 260 | Jinmian25 | Huang River | 1998 | P1 |
| 261 | Shiyuan321 | Huang River | 1998 | P2 |
| 262 | Jimian27 | Huang River | 1998 | P1 |
| 263 | Zhong51504 | Huang River | 1998 | P1 |
| 264 | Yumian18 | Huang River | 1998 | P1 |
| 265 | Jimian25 | Huang River | 1998 | P2 |
| 266 | Nannongzao | Yangtze River | 1998 | P2 |
| 267 | Ekangmian8 | Yangtze River | 1998 | P2 |
| 268 | Ekangmian7 | Yangtze River | 1998 | P2 |
| 269 | XiangSC-24 | Yangtze River | 1998 | P2 |
| 270 | Zhongmiansuo9409 | Huang River | 1999 | P2 |
| 271 | Zhongzongxu1 | Huang River | 1999 | P1 |
| 272 | Yumian19 | Huang River | 1999 | P1 |
| 273 | Jinmian28 | Huang River | 1999 | P1 |
| 274 | Shan2234 | Huang River | 1999 | P2 |
| 275 | Yumian20 | Huang River | 1999 | P1 |
| 276 | Zhongmiansuo36 | Huang River | 1999 | P1 |
| 277 | Sumian14 | Huang River | 1999 | P1 |
| 278 | Binmian1 | Huang River | 1999 | P2 |
| 279 | Yumian21 | Huang River | 1999 | P2 |
| 280 | Zhonglvxu1 | Huang River | 1999 | P1 |
| 281 | Liaomian16 | North China | 1999 | P1 |
| 282 | Xinluzao9 | North-west China | 1999 | P1 |
| 283 | Xinluzao11 | North-west China | 1999 | P1 |
| 284 | Xinluzao10 | North-west China | 1999 | P1 |
| 285 | Xinluzhong8 | North-west China | 1999 | P2 |
| 286 | Sumian15 | Yangtze River | 1999 | P2 |
| 287 | Chuanmian239 | Yangtze River | 1999 | P2 |
| 288 | Ekangmian9 | Yangtze River | 1999 | P2 |
| 289 | Sumian16 | Yangtze River | 1999 | P2 |
| 290 | Ekangmian10 | Yangtze River | 1999 | P2 |
| 291 | Zhongmiansuo37 | Huang River | 2000 | P1 |
| 292 | Jinmian29 | Huang River | 2000 | P2 |
| 293 | Liaomian17 | North China | 2000 | P1 |
| 294 | Xinluzao12 | North-west China | 2000 | P1 |
| 295 | Chuanmian45 | Yangtze River | 2000 | P2 |
| 296 | I40007 | Yangtze River | 2000 | P2 |
| 297 | I40006 | Yangtze River | 2000 | P2 |
| 298 | Shumian1 | Yangtze River | 2000 | P2 |
| 299 | Wanmian17 | Yangtze River | 2000 | P2 |
| 300 | SGK321 | Huang River | 2001 | P2 |
| 301 | Yu668 | Huang River | 2001 | P2 |
| 302 | Yumian1 | Yangtze River | 2001 | P2 |
| 303 | Xinyan96-48 | Huang River | 2002 | P2 |
| 304 | Zhongmiansuo41 | Huang River | 2002 | P2 |
| 305 | Zhongmiansuo40 | Huang River | 2002 | P2 |
| 306 | Yumian112 | Huang River | 2002 | P2 |
| 307 | Yuzao73 | Huang River | 2002 | P1 |
| 308 | Handan109 | Huang River | 2002 | P2 |
| 309 | Liaomian18 | North China | 2002 | P1 |
| 310 | Xinluzao13 | North-west China | 2002 | P1 |
| 311 | Sumian20 | Yangtze River | 2002 | P2 |
| 312 | Sumian22 | Yangtze River | 2002 | P2 |
| 313 | Huakangmian1 | Yangtze River | 2002 | P2 |
| 314 | Jinmian36 | Huang River | 2003 | P2 |
| 315 | Zhongmiansuo45 | Huang River | 2003 | P2 |
| 316 | Liaomian19 | North China | 2003 | P1 |
| 317 | Xinluzhong17 | North-west China | 2003 | P2 |
| 318 | Emian23 | Yangtze River | 2003 | P1 |
| 319 | Zhongmiansuo44 | Huang River | 2004 | P2 |
| 320 | Zhongmiansuo49 | Huang River | 2004 | P1 |
| 321 | Jimian298 | Huang River | 2004 | P2 |
| 322 | Jifeng197 | Huang River | 2004 | P1 |
| 323 | Han4849 | Huang River | 2004 | P2 |
| 324 | Xinluzao19 | North-west China | 2004 | P1 |
| 325 | Xinluzao21 | North-west China | 2004 | P1 |
| 326 | Xinluzhong21 | North-west China | 2004 | P2 |
| 327 | Ekangmian5 | Yangtze River | 2004 | P2 |
| 328 | Zhongmiansuo43 | Huang River | 2005 | P2 |
| 329 | Lumianyan16 | Huang River | 2005 | P2 |
| 330 | Zhongmiansuo50 | Huang River | 2005 | P1 |
| 331 | Zhongzhimian2 | Huang River | 2005 | P2 |
| 332 | Jifeng106 | Huang River | 2005 | P2 |
| 333 | GK99-1 | Huang River | 2005 | P2 |
| 334 | Lumianyan18 | Huang River | 2005 | P2 |
| 335 | 9901 | Huang River | 2005 | P2 |
| 336 | P164-2 | Huang River | 2005 | P2 |
| 337 | J150 | Huang River | 2005 | P2 |
| 338 | GK44 | Huang River | 2005 | P2 |
| 339 | Lumianyan21 | Huang River | 2005 | P2 |
| 340 | Xinluzao23 | North-west China | 2005 | P1 |
| 341 | Xinluzao20 | North-west China | 2005 | P1 |
| 342 | Xinluzao22 | North-west China | 2005 | P2 |
| 343 | Xinluzao24 | North-west China | 2005 | P1 |
| 344 | Xinluzao26 | North-west China | 2005 | P2 |
| 345 | Xinluzao28 | North-west China | 2005 | P1 |
| 346 | Xinluzao30 | North-west China | 2005 | P1 |
| 347 | Xinluzao32 | North-west China | 2005 | P1 |
| 348 | Sukang191 | Yangtze River | 2005 | P2 |
| 349 | MD51ne | USA | -- | P2 |
| 350 | Uganda3 | Uganda | CK | P2 |
| 351 | King | USA | CK | P2 |
| 352 | Foster6 | USA | CK | P2 |
| 353 | Stoneville2B | USA | CK | P2 |
| 354 | DPL15 | USA | CK | P2 |
| 355 | DPL16 | USA | CK | P2 |
| 356 | TM-1 | USA | genetic standard | P2 |

^a^ P1 and P2 were subpopulations inferred from the Bayesian model-based program STRUCTURE 2.3 using 66 unlinked or weakly linked SSR markers
