# Supplementary material for: Favorable QTL Alleles for Yield and Its Components Identified by Association Mapping in Chinese Upland Cotton Cultivars
Source: PLoS One. 2013 Dec 26;8(12):e82193. doi: 10.1371/journal.pone.0082193 (PMC3873261; doi:10.1371/journal.pone.0082193)
Supplement: Table S2 — Detailed information of 145 polymorphic SSR markers. (DOC) [file pone.0082193.s002.doc]

**Table S2** Detailed information of 145 polymorphic SSR markers

| **Marker** | **Chromosome** | **Position** | **Allele No.** | **Gene diversity** | **PIC** |
| --- | --- | --- | --- | --- | --- |
| NAU2437 | A01(Chr.1) | 15.248 | 6 | 0.65 | 0.59 |
| NAU3254 | A01(Chr.1) | 33.644 | 3 | 0.54 | 0.43 |
| NAU2741 | A01(Chr.1) | 56.665 | 2 | 0.39 | 0.31 |
| NAU4073 | A01(Chr.1) | 85.025 | 2 | 0.5 | 0.37 |
| NAU2419 | A01(Chr.1) | 95.171 | 3 | 0.44 | 0.36 |
| NAU5780 | A02(Chr.2) | 0 | 2 | 0.19 | 0.17 |
| JESPR304 | A02(Chr.2) | 22.517 | 3 | 0.2 | 0.18 |
| NAU2161 | A03(Chr.3) | 0 | 3 | 0.34 | 0.29 |
| NAU862 | A03(Chr.3) | 9.309 | 2 | 0.35 | 0.29 |
| STV097 | A03(Chr.3) | 32.581 | 2 | 0.24 | 0.21 |
| BNL226 | A03(Chr.3) | 59.288 | 5 | 0.21 | 0.20 |
| NAU6584 | A03(Chr.3) | 74.975 | 2 | 0.46 | 0.35 |
| NAU3016 | A03(Chr.3) | 109.395 | 9 | 0.36 | 0.35 |
| NAU7182 | A04(Chr.4) | 21.466 | 3 | 0.01 | 0.01 |
| NAU3592 | A04(Chr.4) | 118.114 | 3 | 0.66 | 0.58 |
| NAU934 | A05(Chr.5) | 19.421 | 2 | 0.2 | 0.18 |
| NAU3273 | A05(Chr.5) | 37.244 | 2 | 0.07 | 0.06 |
| NAU2561 | A05(Chr.5) | 56.73 | 2 | 0.45 | 0.35 |
| NAU3529 | A05(Chr.5) | 86.053 | 2 | 0.36 | 0.29 |
| NAU797 | A05(Chr.5) | 143.032 | 2 | 0.49 | 0.37 |
| NAU3212 | A05(Chr.5) | 152.459 | 7 | 0.48 | 0.45 |
| NAU3269 | A05(Chr.5) | 182.215 | 2 | 0.48 | 0.37 |
| BNL3452 | A05(Chr.5) | 188.717 | 3 | 0.18 | 0.16 |
| NAU3427 | A06(Chr.6) | 5.598 | 3 | 0.22 | 0.20 |
| BNL3650 | A06(Chr.6) | 43.602 | 5 | 0.47 | 0.42 |
| NAU4946 | A06(Chr.6) | 52.091 | 2 | 0.22 | 0.20 |
| NAU1151 | A06(Chr.6) | 91.071 | 2 | 0.01 | 0.01 |
| BNL2569 | A06(Chr.6) | 112.635 | 2 | 0.3 | 0.25 |
| NAU845 | A07(Chr.7) | 54.589 | 3 | 0.34 | 0.28 |
| NAU3654 | A07(Chr.7) | 74.363 | 2 | 0.5 | 0.37 |
| NAU3793 | A08(Chr.8) | 20.312 | 2 | 0.49 | 0.37 |
| BNL3792 | A08(Chr.8) | 48.733 | 3 | 0.48 | 0.41 |
| NAU1505 | A08(Chr.8) | 71.893 | 5 | 0.73 | 0.68 |
| BNL3255 | A08(Chr.8) | 81.913 | 2 | 0.31 | 0.26 |
| JESPR232 | A08(Chr.8) | 121.312 | 2 | 0.07 | 0.06 |
| Gh486 | A09(Chr.9) | 10.554 | 3 | 0.51 | 0.39 |
| NAU6177 | A09(Chr.9) | 20.2 | 5 | 0.51 | 0.41 |
| JESPR274 | A09(Chr.9) | 42.522 | 4 | 0.57 | 0.47 |
| NAU3052 | A09(Chr.9) | 85.077 | 3 | 0.35 | 0.29 |
| NAU462 | A09(Chr.9) | 118.404 | 3 | 0.48 | 0.38 |
| NAU3467 | A10(Chr.10) | 0 | 2 | 0.41 | 0.33 |
| STV031 | A10(Chr.10) | 12.751 | 3 | 0.49 | 0.38 |
| NAU5166 | A10(Chr.10) | 23.423 | 2 | 0.04 | 0.04 |
| NAU440 | A10(Chr.10) | 42.017 | 2 | 0.05 | 0.05 |
| NAU2935 | A10(Chr.10) | 51.592 | 2 | 0.06 | 0.06 |
| NAU2508 | A10(Chr.10) | 128.028 | 3 | 0.56 | 0.48 |
| NAU980 | A11(Chr.11) | 0 | 4 | 0.3 | 0.27 |
| NAU5192 | A11(Chr.11) | 12.441 | 5 | 0.22 | 0.21 |
| BNL3442 | A11(Chr.11) | 22.99 | 4 | 0.54 | 0.43 |
| JESPR135 | A11(Chr.11) | 55.787 | 2 | 0.04 | 0.04 |
| Gh369 | A11(Chr.11) | 84.701 | 4 | 0.19 | 0.18 |
| BNL1066 | A11(Chr.11) | 134.486 | 5 | 0.18 | 0.17 |
| NAU3390 | A11(Chr.11) | 144.088 | 3 | 0.18 | 0.17 |
| NAU5428 | A11(Chr.11) | 155.464 | 4 | 0.59 | 0.50 |
| NAU429 | A11(Chr.11) | 161.327 | 4 | 0.48 | 0.37 |
| BNL1231 | A11(Chr.11) | 169.199 | 2 | 0.49 | 0.37 |
| NAU4047 | A12(Chr.12) | 11.118 | 2 | 0.45 | 0.35 |
| NAU3561 | A12(Chr.12) | 17.182 | 3 | 0.27 | 0.25 |
| BNL3261 | A12(Chr.12) | 40.276 | 5 | 0.36 | 0.33 |
| NAU2176 | A12(Chr.12) | 70.314 | 2 | 0.01 | 0.01 |
| NAU4020 | A12(Chr.12) | 84.611 | 2 | 0.12 | 0.11 |
| BNL598 | A12(Chr.12) | 117.36 | 5 | 0.3 | 0.27 |
| JESPR300 | A12(Chr.12) | 147.983 | 2 | 0.31 | 0.26 |
| NAU2893 | A13(Chr.13) | 0 | 2 | 0.5 | 0.37 |
| NAU3398 | A13(Chr.13) | 3.311 | 4 | 0.14 | 0.13 |
| NAU2300 | A13(Chr.13) | 48.295 | 3 | 0.02 | 0.02 |
| BNL1707 | A13(Chr.13) | 104.694 | 2 | 0.43 | 0.34 |
| NAU458 | D01(Chr.15) | 0 | 2 | 0.11 | 0.10 |
| BNL2646 | D01(Chr.15) | 75.614 | 3 | 0.21 | 0.19 |
| NAU2901 | D01(Chr.15) | 104.568 | 2 | 0.04 | 0.04 |
| JESPR152 | D01(Chr.15) | 110.252 | 2 | 0.21 | 0.19 |
| NAU1495 | D01(Chr.15) | 117.858 | 2 | 0.04 | 0.04 |
| NAU1070 | D02(Chr.14) | 13.885 | 2 | 0.49 | 0.37 |
| NAU2190 | D02(Chr.14) | 23.957 | 3 | 0.36 | 0.31 |
| NAU3214 | D02(Chr.14) | 31.44 | 3 | 0.48 | 0.37 |
| NAU5027 | D02(Chr.14) | 40.477 | 2 | 0.05 | 0.05 |
| NAU2173 | D02(Chr.14) | 50.341 | 4 | 0.47 | 0.41 |
| NAU5467 | D02(Chr.14) | 90.678 | 2 | 0.49 | 0.37 |
| CIR246 | D02(Chr.14) | 112.473 | 3 | 0.54 | 0.44 |
| NAU3639 | D03(Chr.17) | 29.469 | 2 | 0.49 | 0.37 |
| BNL3590 | D03(Chr.17) | 39.284 | 2 | 0.15 | 0.14 |
| BNL1606 | D03(Chr.17) | 50.883 | 2 | 0.13 | 0.12 |
| NAU5260 | D03(Chr.17) | 73.062 | 3 | 0.48 | 0.37 |
| BNL834 | D03(Chr.17) | 81.098 | 2 | 0.2 | 0.18 |
| BNL4030 | D04(Chr.22) | 0 | 3 | 0.45 | 0.38 |
| NAU3557 | D04(Chr.22) | 19.862 | 3 | 0.43 | 0.36 |
| NAU6966 | D04(Chr.22) | 36.51 | 3 | 0.2 | 0.19 |
| JESPR220 | D04(Chr.22) | 90.931 | 2 | 0.34 | 0.28 |
| NAU3095 | D05(Chr.19) | 10.634 | 3 | 0.56 | 0.48 |
| NAU5005 | D05(Chr.19) | 99.293 | 2 | 0.39 | 0.31 |
| NAU1042 | D05(Chr.19) | 120.77 | 2 | 0.49 | 0.37 |
| JESPR181 | D05(Chr.19) | 139.942 | 3 | 0.23 | 0.21 |
| NAU7024 | D05(Chr.19) | 154.28 | 4 | 0.57 | 0.49 |
| NAU2816 | D05(Chr.19) | 160.89 | 4 | 0.62 | 0.54 |
| NAU2233 | D05(Chr.19) | 171.278 | 3 | 0.48 | 0.37 |
| BNL3594 | D06(Chr.25) | 7.66 | 5 | 0.6 | 0.55 |
| BNL3103 | D06(Chr.25) | 40.595 | 2 | 0.04 | 0.04 |
| TMK19 | D06(Chr.25) | 70.38 | 2 | 0.45 | 0.35 |
| NAU7209 | D06(Chr.25) | 90.955 | 3 | 0.01 | 0.01 |
| BNL3436 | D06(Chr.25) | 112.978 | 2 | 0.35 | 0.29 |
| BNL3359 | D06(Chr.25) | 140.397 | 2 | 0.31 | 0.26 |
| NAU3608 | D07(Chr.16) | 8.182 | 3 | 0.25 | 0.23 |
| NAU3911 | D07(Chr.16) | 28.658 | 2 | 0.23 | 0.21 |
| BNL1604 | D07(Chr.16) | 36.152 | 6 | 0.41 | 0.38 |
| JESPR297 | D07(Chr.16) | 43.109 | 2 | 0.03 | 0.03 |
| BNL1694 | D07(Chr.16) | 52.03 | 3 | 0.49 | 0.40 |
| NAU6752 | D07(Chr.16) | 74.671 | 2 | 0.08 | 0.07 |
| NAU6468 | D07(Chr.16) | 83.101 | 2 | 0.34 | 0.28 |
| NAU493 | D07(Chr.16) | 113.831 | 2 | 0.45 | 0.35 |
| NAU3424 | D07(Chr.16) | 130.127 | 2 | 0.09 | 0.08 |
| CIR388 | D08(Chr.24) | 12.061 | 2 | 0.01 | 0.01 |
| NAU1369 | D08(Chr.24) | 57.708 | 2 | 0.31 | 0.26 |
| NAU478 | D08(Chr.24) | 67.677 | 3 | 0.46 | 0.39 |
| NAU3207 | D08(Chr.24) | 90.625 | 2 | 0.23 | 0.21 |
| NAU1350 | D08(Chr.24) | 108.529 | 2 | 0.03 | 0.03 |
| JESPR291 | D08(Chr.24) | 153.387 | 3 | 0.44 | 0.39 |
| NAU3100 | D09(Chr.23) | 0 | 4 | 0.45 | 0.39 |
| CIR286 | D09(Chr.23) | 17.983 | 4 | 0.3 | 0.27 |
| NAU3986 | D09(Chr.23) | 58.94 | 3 | 0.05 | 0.05 |
| BNL3140 | D09(Chr.23) | 98.539 | 2 | 0.48 | 0.36 |
| JESPR208 | D09(Chr.23) | 118.225 | 2 | 0.36 | 0.30 |
| BNL1414 | D09(Chr.23) | 122.249 | 2 | 0.39 | 0.31 |
| NAU3414 | D09(Chr.23) | 139.801 | 3 | 0.53 | 0.46 |
| NAU453 | D10(Chr.20) | 0 | 4 | 0.28 | 0.25 |
| NAU2776 | D10(Chr.20) | 9.452 | 3 | 0.5 | 0.39 |
| NAU6755 | D10(Chr.20) | 21.926 | 2 | 0.01 | 0.01 |
| NAU3917 | D10(Chr.20) | 31.125 | 2 | 0.03 | 0.03 |
| TML05 | D10(Chr.20) | 50.675 | 3 | 0.49 | 0.37 |
| NAU3368 | D10(Chr.20) | 72.627 | 4 | 0.11 | 0.11 |
| BNL3280 | D10(Chr.20) | 105.444 | 3 | 0.12 | 0.11 |
| BNL1404 | D11(Chr.21) | 33.571 | 2 | 0.04 | 0.04 |
| Gh508 | D11(Chr.21) | 54.48 | 2 | 0.02 | 0.02 |
| NAU5418 | D11(Chr.21) | 75.156 | 3 | 0.61 | 0.52 |
| NAU2361 | D11(Chr.21) | 101.215 | 4 | 0.64 | 0.57 |
| NAU3084 | D12(Chr.26) | 0 | 2 | 0.13 | 0.12 |
| NAU2251 | D12(Chr.26) | 30.229 | 4 | 0.1 | 0.10 |
| BNL3537 | D12(Chr.26) | 39.496 | 2 | 0.34 | 0.28 |
| NAU3862 | D12(Chr.26) | 105.611 | 2 | 0.21 | 0.19 |
| NAU2980 | D13(Chr.18) | 0 | 2 | 0.14 | 0.13 |
| JESPR204 | D13(Chr.18) | 36.478 | 3 | 0.22 | 0.20 |
| NAU3589 | D13(Chr.18) | 46.619 | 3 | 0.34 | 0.29 |
| Gh501 | D13(Chr.18) | 55.386 | 2 | 0.49 | 0.37 |
| NAU2697 | D13(Chr.18) | 85.738 | 2 | 0.42 | 0.33 |
| NAU3011 | D13(Chr.18) | 97 | 3 | 0.5 | 0.38 |
| NAU6582 | D13(Chr.18) | 111.788 | 5 | 0.2 | 0.20 |
