# Supplementary material for: Favorable QTL Alleles for Yield and Its Components Identified by Association Mapping in Chinese Upland Cotton Cultivars
Source: PLoS One. 2013 Dec 26;8(12):e82193. doi: 10.1371/journal.pone.0082193 (PMC3873261; doi:10.1371/journal.pone.0082193)
Supplement: Table S3 — Genetic diversity in different historically released cultivar groups. (DOC) [file pone.0082193.s003.doc]

**Table S3** Genetic diversity in different historically released cultivar groups

| **Groups^a^** | **Accession No.** | **Allele No.** | **Alleles per locus** | **Gene diversity** |
| --- | --- | --- | --- | --- |
| **CK** | 6 | 254 | 1.74 | 0.29 |
| **I** | 26 | 332 | 2.29 | 0.33 |
| **II** | 26 | 312 | 2.15 | 0.29 |
| **III** | 39 | 333 | 2.30 | 0.30 |
| **IV** | 83 | 367 | 2.53 | 0.31 |
| **V** | 125 | 379 | 2.61 | 0.32 |
| **VI** | 49 | 333 | 2.30 | 0.30 |

^a^ CK, I, II, III, IV, V and VI indicate the founder parent group (CK), and the Chinese cultivars released in 1930–1960, 1961–1970, 1971–1980, 1981–1990, 1991–2000 and 2000–2005, respectively
