# Supplementary material for: Favorable QTL Alleles for Yield and Its Components Identified by Association Mapping in Chinese Upland Cotton Cultivars
Source: PLoS One. 2013 Dec 26;8(12):e82193. doi: 10.1371/journal.pone.0082193 (PMC3873261; doi:10.1371/journal.pone.0082193)
Supplement: Table S4 — SSR markers significantly (P≤0.01, −log10 P≥2.0) associated with lint yield traits and their explained phenotypic variation across three different environments. (DOC) [file pone.0082193.s004.doc]

**Table S4** SSR markers significantly (*P*≤0.01, -log_10_*P*≥2.0) associated with fiber yield traits and their explained phenotypic variation across three different environments

| **Traits^a^** | **Marker loci** | **Chr.** | **Position** | **-Log_10_*P*** | | |  | ***R^2^*** | | |
| --- | --- | --- | --- | --- | --- | --- | --- | --- | --- | --- |
|  |  |  |  | **E1** | **E2** | **E3** |  | **E1** | **E2** | **E3** |
| **FY** | NAU6584 | A03(Chr.03) | 74.975 | 2.48 | 2.37 | ns |  | 0.0263 | 0.0293 |  |
|  | NAU3269 | A05(Chr.05) | 182.215 | 3.43 | 4.42 | 3.33 |  | 0.0283 | 0.0460 | 0.0305 |
|  | NAU3427 | A06(Chr.06) | 5.598 | 2.02 | ns | ns |  | 0.0194 |  |  |
|  | NAU4946 | A06(Chr.06) | 52.091 | ns | ns | 2.01 |  |  |  | 0.0165 |
|  | BNL3792 | A08(Chr.08) | 48.733 | ns | 2.89 | ns |  |  | 0.0342 |  |
|  | STV031 | A10(Chr.10) | 12.751 | ns | ns | 2.57 |  |  |  | 0.0347 |
|  | NAU5166 | A10(Chr.10) | 23.423 | ns | 5.49 | ns |  |  | 0.0540 |  |
|  | NAU3917 | A10(Chr.10) | 27.641 | ns | 2.68 | ns |  |  | 0.0310 |  |
|  | NAU2935 | A10(Chr.10) | 51.592 | ns | 4.00 | ns |  |  | 0.0472 |  |
|  | NAU980 | A11(Chr.11) | 0 | ns | ns | 2.85 |  |  |  | 0.0518 |
|  | JESPR135 | A11(Chr.11) | 55.787 | ns | 2.59 | ns |  |  | 0.0228 |  |
|  | NAU4047 | A12(Chr.12) | 11.118 | 2.80 | ns | ns |  | 0.0225 |  |  |
|  | JESPR204 | A13(Chr.13) | 59.714 | 4.12 | 3.00 | ns |  | 0.0460 | 0.0408 |  |
|  | BNL1707 | A13(Chr.13) | 104.694 | 2.62 | ns | ns |  | 0.0198 |  |  |
|  | NAU3214 | D02(Chr.14) | 31.44 | ns | ns | 2.00 |  |  |  | 0.0230 |
|  | CIR246 | D02(Chr.14) | 112.473 | ns | ns | 3.55 |  |  |  | 0.0444 |
|  | BNL3590 | D03(Chr.17) | 39.284 | ns | 2.77 | 3.00 |  |  | 0.0328 | 0.0331 |
|  | BNL4030 | D04(Chr.22) | 0 | ns | 2.15 | ns |  |  | 0.0359 |  |
|  | NAU3095 | D05(Chr.19) | 10.634 | 2.06 | ns | ns |  | 0.0210 |  |  |
|  | NAU2233 | D05(Chr.19) | 171.278 | 2.17 | 3.22 | 2.25 |  | 0.0268 | 0.0442 | 0.0301 |
|  | BNL3594 | D06(Chr.25) | 7.66 | ns | 4.51 | ns |  |  | 0.0765 |  |
|  | TMK19 | D06(Chr.25) | 70.38 | 4.30 | 2.77 | 3.97 |  | 0.0421 | 0.0321 | 0.0428 |
|  | BNL1694 | D07(Chr.16) | 52.03 | ns | 2.47 | ns |  |  | 0.0345 |  |
|  | NAU3100 | D09(Chr.23) | 25.262 | 3.67 | 5.38 | 4.31 |  | 0.0408 | 0.0708 | 0.0541 |
|  | JESPR208 | D09(Chr.23) | 118.225 | ns | 2.39 | ns |  |  | 0.0282 |  |
|  | NAU2776 | D10(Chr.20) | 9.452 | ns | 3.81 | 2.54 |  |  | 0.0441 | 0.0276 |
|  | BNL1404 | D11(Chr.21) | 33.571 | ns | 2.10 | ns |  |  | 0.0178 |  |
| **SY** | NAU6584 | A03(Chr.03) | 74.975 | 2.09 | 2.59 | ns |  | 0.0234 | 0.0327 |  |
|  | NAU3269 | A05(Chr.05) | 182.215 | 2.25 | 4.16 | 2.43 |  | 0.0185 | 0.0441 | 0.0219 |
|  | BNL3792 | A08(Chr.08) | 48.733 | ns | 2.96 | ns |  |  | 0.0363 |  |
|  | STV031 | A10(Chr.10) | 12.751 | ns | ns | 2.08 |  |  |  | 0.0297 |
|  | NAU5166 | A10(Chr.10) | 23.423 | ns | 3.25 | ns |  |  | 0.0307 |  |
|  | NAU2935 | A10(Chr.10) | 51.592 | ns | 2.92 | ns |  |  | 0.0356 |  |
|  | NAU4047 | A12(Chr.12) | 11.118 | 2.28 | ns | ns |  | 0.0187 |  |  |
|  | JESPR204 | A13(Chr.13) | 59.714 | 2.80 | 2.82 | ns |  | 0.0346 | 0.0401 |  |
|  | BNL1707 | A13(Chr.13) | 104.694 | 3.01 | ns | ns |  | 0.0248 |  |  |
|  | NAU458 | D01(Chr.15) | 0 | ns | 2.04 | ns |  |  | 0.0189 |  |
|  | CIR246 | D02(Chr.14) | 112.473 | ns | ns | 4.23 |  |  |  | 0.0536 |
|  | BNL3590 | D03(Chr.17) | 39.284 | ns | 2.96 | 2.11 |  |  | 0.0361 | 0.0244 |
|  | NAU2233 | D05(Chr.19) | 171.278 | ns | 3.00 | 1.83 |  |  | 0.0425 | 0.0260 |
|  | BNL3594 | D06(Chr.25) | 7.66 | ns | 4.61 | ns |  |  | 0.0797 |  |
|  | TMK19 | D06(Chr.25) | 70.38 | 2.77 | ns | 2.72 |  | 0.0291 |  | 0.0306 |
|  | BNL1694 | D07(Chr.16) | 52.03 | ns | 3.06 | ns |  |  | 0.0428 |  |
|  | NAU3100 | D09(Chr.23) | 25.262 | ns | 4.03 | 2.82 |  |  | 0.0561 | 0.0384 |
|  | JESPR208 | D09(Chr.23) | 118.225 | ns | 2.03 | ns |  |  | 0.0246 |  |
|  | NAU2776 | D10(Chr.20) | 9.452 | ns | 2.46 | 2.04 |  |  | 0.0295 | 0.0230 |
|  | NAU6755 | D10(Chr.20) | 21.926 | ns | 2.15 | ns |  |  | 0.0188 |  |
| **BN** | NAU6584 | A03(Chr.03) | 74.975 | 3.53 | 2.29 | ns |  | 0.0393 | 0.0291 |  |
|  | NAU3269 | A05(Chr.05) | 182.215 | 2.08 | 3.52 | 3.45 |  | 0.0174 | 0.0364 | 0.0344 |
|  | NAU845 | A07(Chr.07) | 54.589 | ns | 2.06 | ns |  |  | 0.0301 |  |
|  | NAU3793 | A08(Chr.08) | 20.312 | ns | ns | 2.34 |  |  |  | 0.0310 |
|  | BNL3792 | A08(Chr.08) | 48.733 | ns | 2.04 | ns |  |  | 0.0250 |  |
|  | NAU1369 | A08(Chr.08) | 90.223 | 2.34 | ns | ns |  | 0.0252 |  |  |
|  | NAU5166 | A10(Chr.10) | 23.423 | ns | 2.34 | ns |  |  | 0.0208 |  |
|  | NAU2935 | A10(Chr.10) | 51.592 | ns | 2.82 | ns |  |  | 0.0343 |  |
|  | NAU1151 | A12(Chr.12) | 97.965 | 2.85 | ns | ns |  | 0.0230 |  |  |
|  | JESPR204 | A13(Chr.13) | 59.714 | 2.40 | 3.12 | ns |  | 0.0302 | 0.0435 |  |
|  | BNL2646 | D01(Chr.15) | 75.614 | ns | 2.01 | ns |  |  | 0.0301 |  |
|  | CIR246 | D02(Chr.14) | 112.473 | ns | ns | 2.82 |  |  |  | 0.0391 |
|  | NAU7024 | D03(Chr.17) | 17.081 | ns | 2.24 | ns |  |  | 0.0328 |  |
|  | BNL3590 | D03(Chr.17) | 39.284 | ns | 2.19 | ns |  |  | 0.0269 |  |
|  | NAU2233 | D05(Chr.19) | 171.278 | ns | 2.30 | ns |  |  | 0.0338 |  |
|  | BNL3594 | D06(Chr.25) | 7.66 | ns | 3.62 | ns |  |  | 0.0667 |  |
|  | TMK19 | D06(Chr.25) | 70.38 | 3.58 | ns | 2.41 |  | 0.0371 |  | 0.0284 |
|  | BNL3359 | D06(Chr.25) | 140.397 | 2.09 | ns | ns |  | 0.0231 |  |  |
|  | NAU493 | D07(Chr.16) | 113.413 | 3.91 | ns | ns |  | 0.0338 |  |  |
|  | NAU3100 | D09(Chr.23) | 25.262 | ns | 3.48 | ns |  |  | 0.0492 |  |
|  | NAU6582 | D13(Chr.18) | 111.788 | 2.00 | ns | ns |  | 0.0302 |  |  |
| **BW** | NAU7049 | A01(Chr.1) | 36.729 | 2.33 | ns | ns |  | 0.0278 |  |  |
|  | JESPR304 | A02(Chr.02) | 22.517 | 2.68 | ns | ns |  | 0.0381 |  |  |
|  | NAU3016 | A03(Chr.03) | 109.395 | ns | ns | 3.00 |  |  |  | 0.0675 |
|  | BNL3452 | A05(Chr.05) | 188.717 | 2.37 | ns | ns |  | 0.0352 |  |  |
|  | BNL3255 | A08(Chr.08) | 81.913 | 2.47 | ns | ns |  | 0.0306 |  |  |
|  | BNL1414 | A09(Chr.09) | 95.911 | ns | 3.26 | 3.72 |  |  | 0.0418 | 0.0445 |
|  | NAU980 | A11(Chr.11) | 0 | 3.13 | 2.54 | ns |  | 0.0623 | 0.0572 |  |
|  | JESPR135 | A11(Chr.11) | 55.787 | ns | 2.82 | ns |  |  | 0.0279 |  |
|  | NAU4047 | A12(Chr.12) | 11.118 | 3.29 | 4.17 | ns |  | 0.0332 | 0.0461 |  |
|  | NAU3398 | A13(Chr.13) | 3.311 | 3.47 | 3.46 | ns |  | 0.0565 | 0.0603 |  |
|  | JESPR204 | A13(Chr.13) | 59.714 | 2.11 | ns | ns |  | 0.0310 |  |  |
|  | CIR246 | D02(Chr.14) | 112.473 | ns | ns | 2.01 |  |  |  | 0.0297 |
|  | BNL3590 | D03(Chr.17) | 39.284 | ns | 2.28 | ns |  |  | 0.0302 |  |
|  | BNL4030 | D04(Chr.22) | 0 | ns | 2.27 | ns |  |  | 0.0411 |  |
|  | NAU3557 | D04(Chr.22) | 19.862 | ns | ns | 2.60 |  |  |  | 0.0332 |
|  | JESPR220 | D04(Chr.22) | 90.931 | ns | 2.44 | ns |  |  | 0.0326 |  |
|  | NAU5005 | D05(Chr.19) | 99.293 | ns | 3.12 | ns |  |  | 0.0404 |  |
|  | NAU2816 | D05(Chr.19) | 160.89 | ns | 2.80 | ns |  |  | 0.0436 |  |
|  | NAU2233 | D05(Chr.19) | 171.278 | ns | 2.77 | ns |  |  | 0.0429 |  |
|  | BNL1694 | D07(Chr.16) | 52.03 | ns | 2.14 | ns |  |  | 0.0337 |  |
|  | JESPR208 | D09(Chr.23) | 118.225 | ns | 4.01 | 3.69 |  |  | 0.0515 | 0.0445 |
|  | BNL3280 | D10(Chr.20) | 105.444 | 2.72 | ns | ns |  | 0.0323 |  |  |
|  | BNL1404 | D11(Chr.21) | 33.571 | ns | 2.27 | ns |  |  | 0.0215 |  |
|  | NAU2251 | D12(Chr.26) | 30.229 | ns | ns | 2.12 |  |  |  | 0.0316 |
|  | NAU3589 | D13(Chr.18) | 46.619 | 2.24 | ns | ns |  | 0.0280 |  |  |
| **LP** | NAU2437 | A01(Chr.01) | 15.248 | ns | ns | 2.30 |  |  |  | 0.0467 |
|  | JESPR304 | A02(Chr.02) | 22.517 | 3.12 | ns | 2.25 |  | 0.0378 |  | 0.0315 |
|  | NAU3269 | A05(Chr.05) | 182.215 | 3.54 | 2.21 | 2.72 |  | 0.0318 | 0.0202 | 0.0259 |
|  | NAU845 | A07(Chr.07) | 54.589 | 2.52 | ns | ns |  | 0.0311 |  |  |
|  | BNL3792 | A08(Chr.08) | 48.733 | ns | ns | 2.00 |  |  |  | 0.0234 |
|  | STV031 | A10(Chr.10) | 12.751 | ns | 2.27 | ns |  |  | 0.0327 |  |
|  | NAU5166 | A10(Chr.10) | 23.423 | 2.33 | 4.36 | 2.74 |  | 0.0180 | 0.0410 | 0.0244 |
|  | NAU2935 | A10(Chr.10) | 51.592 | ns | 2.21 | 2.09 |  |  | 0.0259 | 0.0245 |
|  | NAU2508 | A10(Chr.10) | 128.028 | 4.45 | 3.62 | 4.68 |  | 0.0523 | 0.0481 | 0.0609 |
|  | NAU980 | A11(Chr.11) | 0 | 3.40 | ns | 3.75 |  | 0.0565 |  | 0.0672 |
|  | JESPR135 | A11(Chr.11) | 55.787 | 3.79 | 6.64 | 3.80 |  | 0.0316 | 0.0645 | 0.0354 |
|  | BNL1066 | A11(Chr.11) | 134.486 | ns | 2.52 | ns |  |  | 0.0400 |  |
|  | NAU4020 | A12(Chr.12) | 84.611 | 2.03 | 2.68 | ns |  | 0.0152 | 0.0234 |  |
|  | NAU3398 | A13(Chr.13) | 3.311 | 3.50 | ns | ns |  | 0.0499 |  |  |
|  | JESPR204 | A13(Chr.13) | 59.714 | 3.73 | 2.96 | 2.22 |  | 0.0441 | 0.0396 | 0.0311 |
|  | NAU1070 | D02(Chr.14) | 13.885 | ns | ns | 2.20 |  |  |  | 0.0263 |
|  | CIR246 | D02(Chr.14) | 112.473 | 2.28 | ns | ns |  | 0.0285 |  |  |
|  | BNL3590 | D03(Chr.17) | 39.284 | 4.70 | ns | 2.89 |  | 0.0491 |  | 0.0338 |
|  | JESPR220 | D04(Chr.22) | 90.931 | 2.66 | ns | ns |  | 0.0285 |  |  |
|  | NAU3095 | D05(Chr.19) | 10.634 | ns | ns | 2.22 |  |  |  | 0.0270 |
|  | NAU2233 | D05(Chr.19) | 171.278 | 2.70 | ns | ns |  | 0.0337 |  |  |
|  | BNL3594 | D06(Chr.25) | 7.66 | 2.11 | 2.12 | 2.19 |  | 0.0396 | 0.0437 | 0.0451 |
|  | BNL3103 | D06(Chr.25) | 40.595 | ns | 2.25 | ns |  |  | 0.0259 |  |
|  | TMK19 | D06(Chr.25) | 70.38 | 5.97 | 4.58 | 3.67 |  | 0.0604 | 0.0518 | 0.0419 |
|  | NAU3608 | D07(Chr.16) | 8.182 | 2.06 | 3.04 | ns |  | 0.0216 | 0.0350 |  |
|  | NAU3911 | D07(Chr.16) | 28.658 | 2.43 | ns | ns |  | 0.0207 |  |  |
|  | JESPR297 | D07(Chr.16) | 43.109 | 2.15 | 2.40 | ns |  | 0.0165 | 0.0207 |  |
|  | NAU3100 | D09(Chr.23) | 25.262 | 4.50 | 3.35 | 2.89 |  | 0.0535 | 0.0458 | 0.0400 |
|  | NAU2776 | D10(Chr.20) | 9.452 | ns | 2.02 | ns |  |  | 0.0231 |  |
|  | NAU3917 | D10(Chr.20) | 31.125 | ns | 3.57 | ns |  |  | 0.0403 |  |
|  | NAU3368 | D10(Chr.20) | 72.627 | 2.55 | ns | ns |  | 0.0316 |  |  |
|  | BNL1404 | D11(Chr.21) | 33.571 | 4.48 | 6.20 | 3.51 |  | 0.0381 | 0.0601 | 0.0323 |
|  | Gh508 | D11(Chr.21) | 54.48 | 2.15 | 3.37 | 3.81 |  | 0.0163 | 0.0306 | 0.0354 |
|  | NAU2361 | D11(Chr.21) | 101.215 | 4.05 | 4.30 | 4.03 |  | 0.0637 | 0.0734 | 0.0703 |
|  | NAU3084 | D12(Chr.26) | 0 | 2.64 | ns | ns |  | 0.0212 |  |  |
|  | NAU3589 | D13(Chr.18) | 46.619 | 2.07 | ns | ns |  | 0.0223 |  |  |
| **LI** | NAU2437 | A01(Chr.01) | 15.248 | ns | ns | 2.12 |  |  |  | 0.0465 |
|  | JESPR304 | A02(Chr.02) | 22.517 | 2.82 | ns | 3.26 |  | 0.0365 |  | 0.0456 |
|  | NAU934 | A05(Chr.05) | 19.421 | ns | 2.59 | ns |  |  | 0.0301 |  |
|  | NAU3269 | A05(Chr.05) | 182.215 | 4.98 | 2.77 | 2.38 |  | 0.0492 | 0.0263 | 0.0232 |
|  | BNL3452 | A05(Chr.05) | 188.717 | 2.36 | ns | ns |  | 0.0322 |  |  |
|  | BNL2569 | A06(Chr.06) | 112.635 | 2.54 | ns | ns |  | 0.0212 |  |  |
|  | BNL3792 | A08(Chr.08) | 48.733 | 2.18 | 2.12 | ns |  | 0.0244 | 0.0247 |  |
|  | Gh486 | A09(Chr.09) | 10.554 | 2.27 | ns | ns |  | 0.0363 |  |  |
|  | BNL1414 | A09(Chr.09) | 95.911 | ns | ns | 3.09 |  |  |  | 0.0371 |
|  | STV031 | A10(Chr.10) | 12.751 | ns | 2.77 | 3.44 |  |  | 0.0388 | 0.0496 |
|  | NAU5166 | A10(Chr.10) | 23.423 | ns | 2.96 | 2.08 |  |  | 0.0264 | 0.0183 |
|  | NAU440 | A10(Chr.10) | 42.017 | ns | ns | 2.06 |  |  |  | 0.0180 |
|  | NAU2508 | A10(Chr.10) | 128.028 | 2.59 | ns | 2.01 |  | 0.0341 |  | 0.0304 |
|  | NAU980 | A11(Chr.11) | 0 | 8.85 | 2.34 | 2.80 |  | 0.0896 | 0.0476 | 0.0572 |
|  | JESPR135 | A11(Chr.11) | 55.787 | ns | 5.43 | 2.34 |  |  | 0.0520 | 0.0209 |
|  | Gh369 | A11(Chr.11) | 84.701 | 3.77 | 2.09 | ns |  | 0.0479 | 0.0300 |  |
|  | BNL1066 | A11(Chr.11) | 134.486 | 2.40 | 2.85 | ns |  | 0.0368 | 0.0440 |  |
|  | NAU4047 | A12(Chr.12) | 11.118 | 2.04 | ns | ns |  | 0.0170 |  |  |
|  | NAU4020 | A12(Chr.12) | 84.611 | 2.07 | ns | ns |  | 0.0164 |  |  |
|  | NAU1151 | A12(Chr.12) | 97.965 | 6.08 | ns | ns |  | 0.0563 |  |  |
|  | NAU3398 | A13(Chr.13) | 3.311 | 6.01 | 2.49 | ns |  | 0.0819 | 0.0415 |  |
|  | JESPR204 | A13(Chr.13) | 59.714 | ns | 3.68 | ns |  |  | 0.0481 |  |
|  | JESPR152 | D01(Chr.15) | 110.252 | ns | ns | 2.62 |  |  |  | 0.0314 |
|  | NAU3214 | D02(Chr.14) | 31.44 | ns | ns | 2.64 |  |  |  | 0.0338 |
|  | CIR246 | D02(Chr.14) | 112.473 | 4.75 | 3.14 | ns |  | 0.0577 | 0.0417 |  |
|  | BNL3590 | D03(Chr.17) | 39.284 | 3.30 | 2.70 | 3.47 |  | 0.0367 | 0.0313 | 0.0426 |
|  | BNL1606 | D03(Chr.17) | 50.883 | ns | 3.04 | 2.05 |  |  | 0.0345 | 0.0248 |
|  | NAU3557 | D04(Chr.22) | 19.862 | ns | ns | 2.26 |  |  |  | 0.0290 |
|  | JESPR220 | D04(Chr.22) | 90.931 | 3.11 | ns | ns |  | 0.0350 |  |  |
|  | NAU2816 | D05(Chr.19) | 160.89 | ns | ns | 2.24 |  |  |  | 0.0337 |
|  | NAU2233 | D05(Chr.19) | 171.278 | 4.86 | 2.15 | ns |  | 0.0596 | 0.0304 |  |
|  | BNL3594 | D06(Chr.25) | 7.66 | 2.92 | 2.34 | 3.23 |  | 0.0528 | 0.0469 | 0.0623 |
|  | TMK19 | D06(Chr.25) | 70.38 | 4.83 | 4.02 | ns |  | 0.0519 | 0.0455 |  |
|  | NAU3608 | D07(Chr.16) | 8.182 | 2.92 | 2.38 | ns |  | 0.0322 | 0.0275 |  |
|  | JESPR297 | D07(Chr.16) | 43.109 | 2.26 | ns | ns |  | 0.0185 |  |  |
|  | NAU6752 | D07(Chr.16) | 74.671 | ns | ns | 2.68 |  |  |  | 0.0250 |
|  | NAU3100 | D09(Chr.23) | 25.262 | 5.90 | 2.74 | 3.47 |  | 0.0719 | 0.0384 | 0.0493 |
|  | JESPR208 | D09(Chr.23) | 118.225 | ns | ns | 2.23 |  |  |  | 0.0273 |
|  | NAU2776 | D10(Chr.20) | 9.452 | ns | ns | 3.57 |  |  |  | 0.0430 |
|  | NAU3917 | D10(Chr.20) | 31.125 | ns | 3.66 | 2.03 |  |  | 0.0413 | 0.0245 |
|  | NAU3368 | D10(Chr.20) | 72.627 | 2.08 | ns | ns |  | 0.0279 |  |  |
|  | BNL1404 | D11(Chr.21) | 33.571 | ns | 5.85 | 2.72 |  |  | 0.0564 | 0.0250 |
|  | Gh508 | D11(Chr.21) | 54.48 | ns | 3.67 | 3.41 |  |  | 0.0338 | 0.0327 |
|  | NAU2361 | D11(Chr.21) | 101.215 | 6.27 | 5.16 | 4.58 |  | 0.0940 | 0.0845 | 0.0812 |
|  | NAU3084 | D12(Chr.26) | 0 | 2.00 | ns | ns |  | 0.0161 |  |  |
|  | NAU3589 | D13(Chr.18) | 46.619 | 3.35 | ns | ns |  | 0.0380 |  |  |
|  | NAU6582 | D13(Chr.18) | 111.788 | ns | 2.57 | ns |  |  | 0.0408 |  |
| **SI** | NAU7049 | A01(Chr.1) | 36.729 | 2.52 | ns | ns |  | 0.0319 |  |  |
|  | JESPR304 | A02(Chr.02) | 22.517 | 3.20 | ns | ns |  | 0.0472 |  |  |
|  | NAU3273 | A05(Chr.05) | 37.244 | ns | 2.04 | ns |  |  | 0.0191 |  |
|  | NAU6177 | A09(Chr.09) | 20.2 | ns | 2.06 | ns |  |  | 0.0445 |  |
|  | BNL1414 | A09(Chr.09) | 95.911 | ns | ns | 3.34 |  |  |  | 0.0428 |
|  | NAU1151 | A12(Chr.12) | 97.965 | 2.48 | ns | ns |  | 0.0238 |  |  |
|  | NAU2901 | D01(Chr.15) | 104.568 | 2.07 | ns | ns |  | 0.0191 |  |  |
|  | NAU1495 | D01(Chr.15) | 117.858 | ns | 2.89 | 2.85 |  |  | 0.0370 | 0.0364 |
|  | BNL1606 | D03(Chr.17) | 50.883 | 2.20 | 2.96 | 2.89 |  | 0.0278 | 0.0380 | 0.0366 |
|  | NAU3557 | D04(Chr.22) | 19.862 | ns | ns | 2.80 |  |  |  | 0.0380 |
|  | TMK19 | D06(Chr.25) | 70.38 | ns | ns | 2.01 |  |  |  | 0.0260 |
|  | NAU6752 | D07(Chr.16) | 74.671 | ns | ns | 3.03 |  |  |  | 0.0304 |
|  | NAU493 | D07(Chr.16) | 113.413 | 3.70 | 2.85 | 5.24 |  | 0.0385 | 0.0290 | 0.0575 |
|  | NAU478 | D08(Chr.24) | 67.677 | 3.25 | 2.05 | ns |  | 0.0563 | 0.0396 |  |
|  | NAU3207 | D08(Chr.24) | 90.625 | 2.68 | ns | ns |  | 0.0264 |  |  |
|  | JESPR208 | D09(Chr.23) | 118.225 | ns | ns | 2.04 |  |  |  | 0.0265 |
|  | NAU3084 | D12(Chr.26) | 0 | ns | ns | 2.11 |  |  |  | 0.0200 |
|  | NAU2697 | D13(Chr.18) | 85.738 | ns | 2.05 | ns |  |  | 0.0269 |  |
|  | NAU3011 | D13(Chr.18) | 97 | ns | ns | 2.30 |  |  |  | 0.0360 |

^a^ LY: lint yield (g/plant); SY: seed cotton yield (g/plant); BN: bolls per plant; BW: boll weight (g); LP: lint percentage (%); LI: lint index (g/100 seeds); SI: seed index (g/100 seeds)

^b^ E1, E2, and E3 indicate Jiangpu in 2009, Dafeng in 2010 and Zhengzhou in 2010, respectively
